# Picture fuzzy WASPAS method for selecting last-mile delivery mode: a case study of Belgrade

**DOI:** 10.1186/s12544-021-00501-6

**Published:** 2021-07-30

**Authors:** Vladimir Simić, Dragan Lazarević, Momčilo Dobrodolac

**Affiliations:** Department of Postal and Telecommunication Traffic, Faculty of Transport and Traffic Engineering, University of Belgrade, Vojvode Stepe 305, Belgrade, 11010 Serbia

**Keywords:** Last-mile delivery, Multi-criteria decision-making, Picture fuzzy set, WASPAS, Uncertainty

## Abstract

**Background:**

Last-mile delivery (LMD) is becoming more and more demanding due to an increasing number of users and traffic problems in cities. Besides, medical crises (like the COVID-19 outbreak) and air pollution represent additional motives for the transition from traditional to socially and environmentally sustainable LMD mode. An emerging problem for companies in the postal and logistics industry is how to determine the best LMD mode in a multi-criteria setting under uncertainty.

**Method:**

For the first time, an extension of the Weighted Aggregated Sum Product ASsessment (WASPAS) method under the picture fuzzy environment is presented to solve the LMD mode selection problem. The introduced picture fuzzy set (PFS) based multi-criteria decision-making (MCDM) method can be highly beneficial to managers who are in charge of LMD since it can take into account the neutral/refusal information and efficiently deal with high levels of imprecise, vague, and uncertain information. The comparative analysis with the existing state-of-the-art PFS-based MCDM methods approved the high reliability of the proposed picture fuzzy WASPAS method. Its high robustness and consistency are also confirmed. The presented method can be used to improve LMD in urban areas worldwide. Besides, it can be applied to solve other emerging MCDM problems in an uncertain environment.

**Findings:**

A real-life case study of Belgrade is presented to fully illustrate the potentials and applicability of the picture fuzzy WASPAS method. The results show that postomates are the best mode for LMD in Belgrade, followed by cargo bicycles, drones, traditional delivery, autonomous vehicles, and tube transport.

**Supplementary Information:**

The online version contains supplementary material available at 10.1186/s12544-021-00501-6.

## Introduction

Today’s society, through the needs of different entities, represents a source of numerous new requirements and expectations for companies in the postal and logistics industry. The distribution of different types of shipments represents one of the major needs for these companies. Additional global importance of the delivery system has been particularly apparent during the COVID-19 (coronavirus) outbreak when a large part of the world population encountered some level of restricted movement and social interactions.

In addition to traditional transportation flows, e-commerce is one of the largest generators of shipments of various characteristics [1, 24]. One of the most demanding segments of the delivery process is the last-mile delivery (LMD). It involves the final stage of delivering items to destinations [67]. LMD is one of the most expensive and complex segments of the transportation process [4, 40]. Large distribution systems have some negative impacts, such as environmental pollution or traffic congestions in cities. Besides, the COVID-19 outbreak has created serious disruptions [37]. It resulted in an increasing number of shipments for approximately 45% [47], delays, and lower service quality of LMD [6]. As a result, various LMD modes have emerged such as cargo bicycles, postomates (i.e., locker systems to pick up/deposit parcels), drones, autonomous vehicles, tube transport, etc. These technical solutions can greatly contribute to mitigating these problems.

An emerging problem for companies in the postal and logistics industry is how to determine the best LMD mode from the finite set of available alternatives under numerous conflicting criteria. LMD mode selection is a complex multi-criteria decision-making (MCDM) problem and multiple experts need to participate. An MCDM approach is powerful and flexible for solving logistics and transportation problems [26, 28]. The experts who participate in the selection procedure need to deal with high levels of imprecise, vague, and uncertain information. However, this issue is not fully addressed in the available studies. Besides, the experts may not be entirely familiar with the characteristics of all LMD modes and evaluation criteria due to their distinct position, experience, and education. For that reason, they could abstain or refuse to express their preferences over LMD modes and evaluation criteria. Unfortunately, the present models for the LMD do not take into account the neutral/refusal information.

Picture fuzzy sets (PFSs) [8, 9] are a new mathematical tool for modeling the ambiguous and imprecise information of complicated phenomena and events [14]. This novel extension of fuzzy sets shows superiority in describing decision-maker preferences [34] and mitigating information loss [64]. PFSs are characterized by degrees of positive, neutral, negative, and refusal membership [3, 50]. These advanced fuzzy sets are considerably more close to human nature [56]. For these reasons, this study will utilize PFSs to practically and accurately present uncertainty and vagueness in the LMD mode selection problem.

The Weighted Aggregated Sum Product ASsessment (WASPAS) is one of the newest MCDM methods introduced by Zavadskas et al. [76]. It is a combination of the Weighted sum model (WSM) [17] and the Weighted product model (WPM) [5]. The ranking of alternatives is based on joint generalized criteria value calculated from the outcomes of the WSM and WMP models. The WASPAS method has high efficiency and effectiveness in the process of decision-making [20, 79]. Its main advantages are computational easiness, consistency of results, and strong resistance against rank reversal of alternatives [7, 74]. Despite all its advantages, the WASPAS method is unable to handle imprecise, vague, and uncertain information. Besides, it does not take into account the neutral/refusal information of decision-makers.

This study introduces the picture fuzzy WASPAS method for selecting LMD mode. The developed PFS-based MCDM method is applied to the real-life LMD mode selection problem in the Belgrade scenario. Four main criteria and 19 sub-criteria for assessing LMD modes are distinguished. The three-level hierarchical structure is constructed to offer a practical framework for solving the outlined emerging problem. The robustness of results is checked by performing two sensitivity analyses. The reliability of results is tested by performing comparative analysis with the existing state-of-the-art picture fuzzy MCDM methods. The consistency of results is investigated by applying appropriate quantitative metrics.

The contributions of this study to the present body of knowledge are as follows:

i. For the first time, an extension of the WASPAS method under the picture fuzzy environment is presented. This novel PFS-based method is used to solve the highlighted problem in the multi-criteria group decision-making context under uncertainty.
ii. Unlike other decision-making approaches for LMD, this study uses PFSs to more efficiently present uncertainties and mitigate information loss. Besides, the voting mechanism is implemented into the formulated WASPAS-based methodological framework to allow decision-makers to more naturally express their preferences over LMD modes as well as evaluation criteria and sub-criteria.
iii. A case study of Belgrade provides decision-making guidelines on how to identify the best LMD mode in the real-world context. Its findings indicate the effectiveness of the introduced picture fuzzy WASPAS method.
iv. Although this study aims to improve LMD in urban areas worldwide, the formulated method can be applied to solve other MCDM problems under an uncertain environment.

The rest of the paper is organized as follows: Section 2 provides a review of related research. Section 3 presents the developed picture fuzzy WASPAS method. A real-life case study is described in Section 4. Section 5 presents the case study results and discussions. Section 6 presents the conclusions of the work.

## Literature review

The literature review is organized into three subsections. The first subsection provides a review of available PFS-based MCDM models. The second subsection overviews the available applications of the WASPAS method. The last sub-section presents identified research gaps.

### Picture fuzzy set based multi-criteria decision-making models

Previously, PFS-based MCDM models, in which PFSs are utilized in defining decision-maker linguistic judgments, have been applied in many areas (Table 1).

**Table 1:** Summary of the available picture fuzzy set-based MCDM approaches

Author(s) and year	Research focus	Method	MCGDM	Application type
Wei [68]	Emerging technology commercialization	PF Cross-entropy	No	Illustrative example
Liang et al. [35]	Gold mines	PF EDAS + ELECTRE III	Yes	Illustrative example
Wang et al. [64]	Energy performance contracting	PF MABAC	Yes	Real-life
Wang et al. [66]	Risk evaluation of construction project	PF Entropy + VIKOR	Yes	Real-life
Wei [69]	Emerging technology commercialization	PF TODIM	No	Illustrative example
Wei et al. [70]	Emerging technology commercialization	PF Projection	No	Illustrative example
Zhang et al. [78]	Offshore wind power station location	PF RP + TOPSIS	Yes	Real-life
Ashraf et al. [2]	Air quality	PF TOPSIS	Yes	Illustrative example
Ju et al. [27]	Electric vehicle charging station location	Fuzzy AHP + PF GRP	Yes	Real-life
Liang et al. [34]	Gold mines	PF TODIM + ELECTRE	Yes	Real-life
Liu et al. [36]	Emerging technology commercialization	PF GRA	No	Illustrative example
Meksavang et al. [38]	Beef supply chain	PF VIKOR	Yes	Illustrative example
Sindhu et al. [51]	–	LP + PF TOPSIS	No	Illustrative example
Torun and Gördebil [60]	Satisfaction level	PF TOPSIS	No	Real-life
Zhang et al. [77]	Green supplier selection	PF EDAS	Yes	Illustrative example
Joshi [25]	Personnel and investment selection	PF Entropy + VIKOR	Yes	Illustrative examples
Tian and Peng [57]	Tourism attraction recommendation	PF ANP + TODIM	Yes	Illustrative example
Tian et al. [58]	Tourism attraction recommendation	PF AHP + PROMETHEE II	Yes	Illustrative example
Wang et al. [65]	Hotel selection	PF TODIM	Yes	Real-life
Yue [73]	Software reliability	PF VIKOR	Yes	Illustrative example
*Our study*	*Last-mile delivery mode selection*	*PF WASPAS*	*Yes*	*Real-life*

*GRA* Grey Relational Analysis, *GRP* Grey Relational Projection, *LP* Linear Programming, *MCGDM* Multi-Criteria Group Decision-Making, *PF* Picture Fuzzy, *RP* Relative Projection

Wei [68] developed a picture fuzzy Cross-entropy approach to rank emerging technology enterprises. Liang et al. [35] integrated the EDAS and ELECTRE III methods into the picture fuzzy environment to evaluate cleaner production technologies in gold mines. A combination of the SWARA and picture fuzzy mean-squared deviation approaches was used to determine criteria weights. Wang et al. [64] coupled the picture fuzzy entropy and VIKOR methods to assess risks of construction projects. Wang et al. [66] integrated a modified maximizing deviation method and a picture fuzzy MABAC method to rank risk factors of energy performance contracting projects. Wei [69] proposed a picture fuzzy TODIM method to evaluate emerging technology commercialization alternatives. Wei et al. [70] presented a picture fuzzy projection method to deal with emerging technology commercialization evaluation. Zhang et al. [78] introduced picture fuzzy relative projection models to measure similarities and weights of different decision-maker groups. They applied the picture fuzzy TOPSIS method to evaluate offshore wind power station locations.

Ashraf et al. [2] extended the TOPSIS method into the picture fuzzy environment. Ju et al. [27] proposed a picture fuzzy Grey relational projection method to locate electric vehicle charging stations. The fuzzy AHP method was used to determine criteria weights. Liang et al. [34] combined the TODIM and ELECTRE methods with picture fuzzy information to rank gold mines. The best-worst method and entropy measure were applied to define subjective and objective criteria weights, respectively. Liu et al. [36] proposed a picture fuzzy grey relational analysis, which can handle the cases where criteria weight information are partly known or completely unknown. Meksavang et al. [38] utilized the picture fuzzy VIKOR method to evaluate suppliers in the beef supply chain. Sindhu et al. [51] coupled the linear programming approach and the picture fuzzy TOPSIS method. Torun and Gördebil [60] applied the picture fuzzy TOPSIS method for evaluating citizens’ satisfaction levels from public services. Zhang et al. [77] applied the picture fuzzy EDAS method for solving the green supplier selection problem.

Recently, Joshi [25] coupled a new R-norm picture fuzzy information measure with the VIKOR method to solve MCDM problems with unknown or partially known criteria weight information. Tian and Peng [57] integrated the ANP and TODIM methods to rank tourism attractions with completely unknown criteria weight information under the picture fuzzy environment. Tian et al. [58] combined the AHP method and a picture fuzzy PROMETHEE II method to assess tourism attractions. Wang et al. [65] used the picture fuzzy TODIM method to investigate the hotel selection problems for different traveler types. Yue [73] applied the picture fuzzy VIKOR method for software reliability assessment.

### Applications of the WASPAS method

The WASPAS method is the utility theory-based approach that relies on multiplicative and additive utility functions. It attracted a large interest of researchers in recent years (Table 2).

**Table 2:** Summary of the available applications of the WASPAS method

Author(s) and year	Research focus	Application type	Parameter type
Zavadskas et al. [75]	Waste incineration plant location	Real-life	Single-valued neutrosophic
Zolfani et al. [80]	Supplier selection	Real-life	Deterministic
Ghorabaee et al. [19]	Green supplier selection	Illustrative example	Interval type-2 fuzzy
Petrović et al. [42]	Waste collection	Real-life	Deterministic
Yazdani et al. [72]	Green supplier selection	Real-life	Deterministic
Ghorabaee et al. [18]	3PL provider selection	Illustrative example	Interval type-2 fuzzy
Khodadadi et al. [29]	Wastewater purification	From literature	Deterministic
Deveci et al. [11]	Car sharing site selection	Real-life	Interval type-2 fuzzy
Sremac et al. [53]	3PL provider selection	Real-life	Rough
Stević et al. [55]	Roundabout location	Real-life	Rough
Dimitrova Stoilova [13]	Rail transportation	Real-life	Deterministic
Gupta et al. [21]	Green supplier selection	Real-life	Fuzzy
Krishankumar et al. [30]	Green supplier selection	From literature	Probabilistic linguistic term
Mishra et al. [39]	Green supplier selection	Illustrative example	Hesitant fuzzy
Pamučar et al. [41]	3PL provider selection	Real-life	Interval rough
Prajapati et al. [43]	Reverse logistics barriers	Real-life	Deterministic
Ren et al. [46]	Electric car charging station	Real-life	Hesitant fuzzy linguistic term
Davoudabadi et al. [10]	Supplier selection	From literature	Interval-valued intuitionistic fuzzy
Dorfeshan and Mousavi [15]	Aircraft maintenance	From literature	Interval type-2 fuzzy
Eghtesadifard et al. [16]	Solid waste landfill location	Real-life	Deterministic
Rani and Mishra [45]	Fuel technology selection	From literature	Q-rung orthopair fuzzy
*Our study*	*Last-mile delivery mode selection*	*Real-life*	*Picture fuzzy*

Zavadskas et al. [75] presented a single-valued neutrosophic WASPAS method for sitting waste incineration plants. Zolfani et al. [80] employed a SWARA-WASPAS approach to solve the supplier selection problem in which alternatives were Nash equilibriums. Ghorabaee et al. [19] presented an interval type-2 fuzzy WASPAS method to solve the green supplier selection problem. Petrović et al. [42] employed the WASPAS method to rank alternative fuels and advanced vehicle technologies for waste collection vehicles. Yazdani et al. [72] used the SWARA-WASPAS approach to solve the green supplier selection problem.

Ghorabaee et al. [18] presented an interval type-2 fuzzy CRITIC-WASPAS method to select a distribution agent for home appliances. Khodadadi et al. [29] applied the SWARA-WASPAS approach for evaluating different oxidation processes for wastewater management. Deveci et al. [11] coupled the interval type-2 fuzzy WASPAS and TOPSIS methods to rank locations for car-sharing stations. Sremac et al. [53] introduced a rough SWARA-WASPAS approach to evaluate dangerous material providers. Stević et al. [55] developed a rough BWM-WASPAS approach to rank roundabout construction locations.

Dimitrova Stoilova [13] examined railway transport markets by using the WASPAS method. Gupta et al. [21] employed a fuzzy AHP-WASPAS approach to assess green suppliers. Krishankumar et al. [30] developed a probabilistic linguistic Statistical variance procedure-WASPAS approach and provided two illustrative examples. Mishra et al. [39] exploited hesitant fuzzy sets and integrated the Exponential entropy, Jensen-Shannon divergence, and WASPAS methods to solve the green supplier selection problem. Pamučar et al. [41] introduced an interval rough BWM-WASPAS approach to evaluate providers for an electronics company. Prajapati et al. [43] employed the SWARA-WASPAS approach to mitigate the impact of reverse logistics implementation barriers in the electrical manufacturing industry. Ren et al. [46] proposed a hesitant fuzzy linguistic SWARA-WASPAS approach to rank electric vehicle charging station sites.

Davoudabadi et al. [10] exploited the concept of knowledge measure of interval-valued intuitionistic fuzzy sets to solve the supplier selection problem. The authors employed a WASPAS-TOPSIS approach to aggregate hybrid weights of experts and overall values of alternatives. Dorfeshan and Mousavi [15] exploited the concept of relative preference relation of interval type-2 fuzzy sets and coupled the WASPAS and MABAC methods to solve the aircraft maintenance planning problem. Eghtesadifard et al. [16] used a DEMATEL-ANP-WASPAS approach for ranking solid waste landfill sites. Rani and Mishra [45] presented a q-rung orthopair fuzzy WASPAS method to deal with the alternative-fuel technology selection problem in fleet operations.

### Research gaps

As can be seen from Tables 1 and 2, the WASPAS method has neither been extended under the PFS environment nor been applied for solving the LMD mode selection problem. Hence, to fill these two major gaps with the aid of the developed picture fuzzy WASPAS method, this paper solves the LMD mode selection problem in the Belgrade scenario.

## Methodology

This section firstly gives the concept and operational laws of PFSs, the picture fuzzy weighted arithmetic average operator, and the two-step defuzzification procedure. Then, the three-phase picture fuzzy WASPAS method is formulated and explained. A detailed flowchart is also provided to additionally increase the clarity of presentation of our innovative PFS-based decision-making methodological framework.

### Picture fuzzy sets

**Definition 1** [8, 9]. Let PFS *A* on a universe *X* is an object in the form of:

1 $$ A=\left\{<x,{\mu}_A(x),{\eta}_A(x),{\upsilon}_A(x)>|\ x\in X\right\}, $$

where μ_A_(*x*)∈[0, 1] is called the degree of positive membership of *x* in *A*; *η*_*A*_(*x*)∈[0, 1] is the degree of neutral membership of *x* in *A*; *υ*_*A*_(*x*)∈[0, 1] is the degree of negative membership of *x* in *A*; and *μ*_*A*_(*x*), *η*_*A*_(*x*), and *υ*_*A*_(*x*) satisfy the following condition:

2 $$ 0\le {\mu}_A(x)+{\eta}_A(x)+{\upsilon}_A(x)\le 1,\kern0.75em \forall x\in X. $$

The word “picture” in PFS refers to generality, as this set is the direct extension of fuzzy sets and intuitionistic fuzzy sets (IFSs). In the case when *η*_*A*_(*x*) = 0, the PFS returns to the IFS. When both *η*_*A*_(*x*) = *υ*_*A*_(*x*) = 0, the PFS returns to the fuzzy set. The integration of the degree of neutral membership *η*_*A*_(*x*) measures the information of objects more accurately and increases the quality and accuracy of achieved results. In PFS theory, decision-makers are divided into four groups: vote for (its ratio is denoted as *μ*), abstain (its ratio is denoted as *η*), vote against (its ratio is denoted as *υ*), and refusal of voting (its ratio is denoted as *ξ*).

The degree of refusal membership of *x* in the PFS *A* can be calculated as follows:

3 $$ {\xi}_A(x)=1-\left({\mu}_A(x)+{\eta}_A(x)+{\upsilon}_A(x)\right),\kern0.75em \forall x\in X. $$

In particular, if *X* has only one element, then *A* = {<*x*, *μ*_*A*_(*x*), *η*_*A*_(*x*), *υ*_*A*_(*x*) > | *x*∈*X*} is called a picture fuzzy number (PFN). For convenience, a PFN is denoted by *A* = <*μ*_*A*_, *η*_*A*_, *υ*_*A*_ > .

**Definition 2** [8, 9]. The complement of a PFS *A* = {<*x*, *μ*_*A*_(*x*), *η*_*A*_(*x*), *υ*_*A*_(*x*) > | *x*∈*X*} on a universe *X* is represented as:

4 $$ {A}^c=\left\{<x,{\upsilon}_A(x),{\eta}_A(x),{\mu}_A(x)>|\ x\in X\right\}. $$

**Definition 3** [35, 63]. Let *A* = <*μ*_*A*_, *η*_*A*_, *υ*_*A*_>, ${A}_1=<{\mu}_{A_1},{\eta}_{A_1},{\upsilon}_{A_1}>,$ and ${A}_2=<{\mu}_{A_2},{\eta}_{A_2},{\upsilon}_{A_2}>$ be three PFNs, and *λ* > 0. Their operations rules are defined as follows:

5 $$ {A}_1\oplus {A}_2=<1-\left(1-{\mu}_{A_1}\right)\left(1-{\mu}_{A_2}\right),{\eta}_{A_1}{\eta}_{A_2},\left({\eta}_{A_1}+{\upsilon}_{A_1}\right)\left({\eta}_{A_2}+{\upsilon}_{A_2}\right)-{\eta}_{A_1}{\eta}_{A_2}>, $$

6 $$ {A}_1\otimes {A}_2=<\left({\mu}_{A_1}+{\eta}_{A_1}\right)\left({\mu}_{A_2}+{\eta}_{A_2}\right)-{\eta}_{A_1}{\eta}_{A_2},{\eta}_{A_1}{\eta}_{A_2},1-\left(1-{\upsilon}_{A_1}\right)\left(1-{\upsilon}_{A_2}\right)>, $$

7 $$ \lambda \cdot A=<1-{\left(1-{\mu}_A\right)}^{\lambda },{\left({\eta}_A\right)}^{\lambda },{\left({\eta}_A+{\upsilon}_A\right)}^{\lambda }-{\left({\eta}_A\right)}^{\lambda }>, $$

8 $$ {A}^{\lambda }=<{\left({\mu}_A+{\eta}_A\right)}^{\lambda }-{\left({\eta}_A\right)}^{\lambda },{\left({\eta}_A\right)}^{\lambda },1-{\left(1-{\upsilon}_A\right)}^{\lambda }>. $$

**Definition 4** [35, 63]. Let ${A}_i=<{\mu}_{A_i},{\eta}_{A_i},{\upsilon}_{A_i}>$ (*i* = 1, ..., *n*) be a collection of PFNs, and *λ* = (*λ*_1_, ..., *λ*_*n*_)^*T*^ be the weight vector of them, with *λ*_*i*_∈[0, 1] and $\sum \limits_{i=1}^n{\lambda}_i=1.$ The picture fuzzy weighted arithmetic average (PFWAA) operator is defined as follows:

9 $$ {\displaystyle \begin{array}{l}{PFWAA}_{\lambda}\left({A}_1,\dots, {A}_n\right)=\underset{i=1}{\overset{n}{\oplus }}\left({\lambda}_i\cdot {A}_i\right)\\ {}=<1-\prod \limits_{i=1}^n{\left(1-{\mu}_{A_i}\right)}^{\lambda_i},\prod \limits_{i=1}^n{\left({\eta}_{A_i}\right)}^{\lambda_i},\prod \limits_{i=1}^n{\left({\eta}_{A_i}+{\upsilon}_{A_i}\right)}^{\lambda_i}-\prod \limits_{i=1}^n{\left({\eta}_{A_i}\right)}^{\lambda_i}>,\end{array}} $$

and the picture fuzzy weighted geometric average (PFWGA) operator is defined as follows:

10 $$ {\displaystyle \begin{array}{l}{PFWGA}_{\lambda}\left({A}_1,\dots, {A}_n\right)=\underset{i=1}{\overset{n}{\otimes }}{\left({A}_i\right)}^{\lambda_i}\\ {}=<\prod \limits_{i=1}^n{\left({\mu}_{A_i}+{\eta}_{A_i}\right)}^{\lambda_i}-\prod \limits_{i=1}^n{\left({\eta}_{A_i}\right)}^{\lambda_i},\prod \limits_{i=1}^n{\left({\eta}_{A_i}\right)}^{\lambda_i},1-\prod \limits_{i=1}^n{\left(1-{\upsilon}_{A_i}\right)}^{\lambda_i}>.\end{array}} $$

**Definition 5** [49, 52]. Let *A* = <*μ*_*A*_, *η*_*A*_, *υ*_*A*_ > be a PFN. The two-step defuzzification procedure to obtain a crisp value of the PFN *A* is:

Step 1. Distribute the neutral degree to the positive and negative degrees as follows:

11 $$ {\mu}_A^{\hbox{'}}={\mu}_A+\frac{\eta_A}{2}, $$

12 $$ {\upsilon}_A^{\hbox{'}}={\upsilon}_A+\frac{\eta_A}{2}. $$

Step 2. Calculate the crisp value *y* by:

13 $$ y={\mu}_A^{\hbox{'}}+\frac{1+{\mu}_A^{\hbox{'}}-{\upsilon}_A^{\hbox{'}}}{2}\xi, $$

where the refusal ratio is defined based on Eq. (3) as *ξ* = 1-(*μ* + *η* + *υ*).

### Picture fuzzy WASPAS method

Let A = {*A*_1_, …, *A*_*m*_} (*m* ≥ 2) be a finite set of alternatives which experts have to choose from, C = {*C*_1_, …, *C*_*n*_} (*n* ≥ 2) be a finite set of criteria with which performances of the alternatives can be measured, ${C}_{s_j}=\left\{{C}_{j1},\dots, {C}_{jn_j}\right\}$ be a finite set of sub-criteria for the *j*-th criterion *C*_*j*_, and D = {*D*_1_, …, *D*_*k*_} (*k* ≥ 2) be a set of invited experts.

**Fig. 1:**
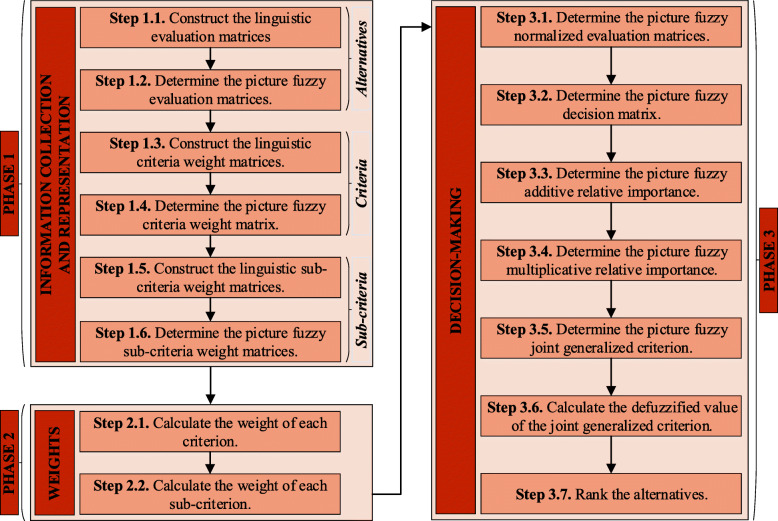
The flowchart of the developed picture fuzzy WASPAS method

The flowchart of the proposed picture fuzzy WASPAS method is presented in Fig. 1. The developed method involves three phases. In phase 1, linguistic importance evaluations are collected and expressed as PFNs. In phase 2, weights of criteria and sub-criteria are determined. In phase 3, the ranking results of the alternatives are obtained.

The details of the phases are given in the following:

***Phase 1:*** *information collection and representation*

**Step 1.1** Construct the linguistic evaluation matrices ${\Gamma}_{ij}={\left[{\gamma}_{{ij s}_j}^e\right]}_{n_j\times k}:$

14 $$ {\displaystyle \begin{array}{l}\kern5.12em {D}_1\kern0.5em \cdots \kern0.74em {D}_k\\ {}{\Gamma}_{ij}=\begin{array}{c}{C}_{j1}\\ {}\vdots \\ {}{C}_{jn_j}\end{array}\left[\begin{array}{ccc}{\gamma}_{ij1}^1& \cdots & {\gamma}_{ij1}^k\\ {}\vdots & \ddots & \vdots \\ {}{\gamma}_{{ij n}_j}^1& \cdots & {\gamma}_{{ij n}_j}^k\end{array}\right],\kern0.75em i=1,\dots, m;j=1,\dots, n,\end{array}} $$

where ${\gamma}_{ijs_j}^e$ is the linguistic evaluation given by the expert *D*_*e*_ towards the alternative *A*_*i*_ (*i* = 1, ..., *m*) with respect to the sub-criterion ${C}_{s_j}$ of the criterion *C*_*j*_. Importance evaluations can be yes, abstain, no, and refusal. Group abstain means that the voting paper is a blank paper rejecting both “yes” and “no” but still takes the vote. Group refusal of voting is either invalid voting papers or did not take the vote.

**Step 1.2** Determine the picture fuzzy evaluation matrices ${Z}_j={\left[{z}_{ijs_j}\right]}_{m\times {n}_j}:$

15 $$ {\displaystyle \begin{array}{l}\kern3.99em {C}_{j1}\kern0.5em \cdots \kern1.00em {C}_{jn_j}\\ {}{Z}_j=\begin{array}{c}{A}_1\\ {}\vdots \\ {}{A}_m\end{array}\left[\begin{array}{ccc}{z}_{1j1}& \cdots & {z}_{1{jn}_j}\\ {}\vdots & \ddots & \vdots \\ {}{z}_{mj1}& \cdots & {z}_{{mj n}_j}\end{array}\right],\kern0.75em j=1,\dots, n,\end{array}} $$

where ${z}_{ijs_j}=<{\mu}_{z_{ijs_j}},{\eta}_{z_{ijs_j}},{\upsilon}_{z_{ijs_j}}>$ is a PFN which represents an evaluation of the alternative *A*_*i*_ with respect to the sub-criterion ${C}_{s_j}$ of the criterion *C*_*j*_ given by the experts. The four types of voting results are fully in accordance with the four components of a PFN. Importance evaluations given by the experts can be expressed as PFNs by calculating the proportion of each item in the voting results.

**Step 1.3** Construct the linguistic criteria weight matrices ${\Psi}^e={\left[{\psi}_j^e\right]}_{n\times 1}:$

16 $$ {\Psi}^e={\displaystyle \begin{array}{c}{C}_1\\ {}\vdots \\ {}{C}_n\end{array}}\left[\begin{array}{c}{\psi}_1^e\\ {}\vdots \\ {}{\psi}_n^e\end{array}\right],\kern0.75em e=1,\dots, k, $$

where ${\psi}_j^e$ is the linguistic importance evaluation given by the expert *D*_*e*_ (*e* = 1, ..., *k*) towards the criterion *C*_*j*_ (*j* = 1, ..., *n*). Importance evaluations towards criteria can be yes, abstain, no, and refusal.

**Step 1.4** Determine the picture fuzzy criteria weight matrix *V* = [*v*_*j*_]_*n* × 1_:

17 $$ V={\displaystyle \begin{array}{c}{C}_1\\ {}\vdots \\ {}{C}_n\end{array}}\left[\begin{array}{c}{v}_1\\ {}\vdots \\ {}{v}_n\end{array}\right], $$

where ${v}_j=<{\mu}_{v_j},{\eta}_{v_j},{\upsilon}_{v_j}>$ is a PFN which represents the importance evaluation of the criterion *C*_*j*_ given by the experts. It is calculated as the proportion of each item in the voting results.

**Step 1.5** Construct the linguistic sub-criteria weight matrices ${\Phi}^e={\left[{\phi}_{js_j}^e\right]}_{n\times 1}:$

18 $$ {\Phi}_j^e={\displaystyle \begin{array}{c}{C}_{j1}\\ {}\vdots \\ {}{C}_{jn_j}\end{array}}\left[\begin{array}{c}{\phi}_{j1}^e\\ {}\vdots \\ {}{\phi}_{jn_j}^e\end{array}\right],\kern0.75em j=1,\dots, n;e=1,\dots, k, $$

where ${\phi}_{js_j}^e$ is the linguistic importance evaluation given by the expert *D*_*e*_ towards the sub-criterion ${C}_{s_j}$ (*s*_*j*_ = 1, ..., *n*_*j*_) of the criterion *C*_*j*_. Importance evaluations towards sub-criteria can be yes, abstain, no, and refusal.

**Step 1.6** Determine the picture fuzzy sub-criteria weight matrices ${O}_j={\left[{o}_{js_j}\right]}_{n_j\times 1}:$

19 $$ {O}_j={\displaystyle \begin{array}{c}{C}_{j1}\\ {}\vdots \\ {}{C}_{jn_j}\end{array}}\left[\begin{array}{c}{o}_{j1}\\ {}\vdots \\ {}{o}_{jn_j}\end{array}\right],\kern0.75em j=1,\dots, n, $$

where ${o}_{js_j}=<{\mu}_{o_{js_j}},{\eta}_{o_{js_j}},{\upsilon}_{o_{js_j}}>$ is a PFN which represents the importance evaluation of the sub-criterion ${C}_{s_j}$ of the criterion *C*_*j*_ given by the experts. It is calculated as the proportion of each item in the voting results.

***Phase 2:*** *determination of criteria and sub-criteria weights*

**Step 2.1** Calculate the weight of each criterion as follows:

20 $$ {w}_j=\frac{\mu_{v_j}+\frac{\eta_{v_j}}{2}+\frac{\xi_{v_j}}{2}\left(1+{\mu}_{v_j}-{\upsilon}_{v_j}\right)}{\sum \limits_{l=1}^n\left[{\mu}_{v_l}+\frac{\eta_{v_l}}{2}+\frac{\xi_{v_l}}{2}\left(1+{\mu}_{v_l}-{\upsilon}_{v_l}\right)\right]},\kern0.75em j=1,\dots, n, $$

where ${v}_j=<{\mu}_{v_j},{\eta}_{v_j},{\upsilon}_{v_j}>$ is a PFN which represents the importance evaluation of the criterion *C*_*j*_ given by the experts. Blank ballot papers are divided into half; i.e., one half for the experts who vote for and one half for the experts who vote against; ${\xi}_{v_j}=1-{\mu}_{v_j}-{\eta}_{v_j}-{\upsilon}_{v_j}$ (*j* = 1, ..., *n*) is the ratio of experts which refuse to provide importance evaluation towards the criterion *C*_*j*_; and *w*_*j*_∈[0, 1] (*j* = 1, ..., *n*), $\sum \limits_{j=1}^n{w}_j=1.$.

**Step 2.2** Calculate the weight of each sub-criterion as follows:

21 $$ {\delta}_{js_j}=\frac{\mu_{o_{js_j}}+\frac{\eta_{o_{js_j}}}{2}+\frac{\xi_{o_{js_j}}}{2}\left(1+{\mu}_{o_{js_j}}-{\upsilon}_{o_{js_j}}\right)}{\sum \limits_{s_l=1}^{n_j}\left[{\mu}_{o_{js_l}}+\frac{\eta_{o_{js_l}}}{2}+\frac{\xi_{o_{js_l}}}{2}\left(1+{\mu}_{o_{js_l}}-{\upsilon}_{o_{js_l}}\right)\right]},\kern0.75em j=1,\dots, n;{s}_j=1,\dots, {n}_j, $$

where ${o}_{js_j}=<{\mu}_{o_{js_j}},{\eta}_{o_{js_j}},{\upsilon}_{o_{js_j}}>$ is a PFN which represents importance evaluation of the sub-criterion ${C}_{s_j}$ of the criterion *C*_*j*_ given by the experts; and ${\delta}_{s_j}\in \left[0,1\right]$ (*s*_*j*_ = 1, ..., *n*_*j*_) and $\sum \limits_{s_j=1}^{n_j}{\delta}_{s_j}=1.$.

***Phase 3:*** *decision-making*

**Step 3.1** Determine the picture fuzzy normalized evaluation matrices ${R}_j={\left[{r}_{ijs_j}\right]}_{m\times {n}_j}$ as follows:

22 $$ {\displaystyle \begin{array}{l}{r}_{ijs_j}=\left\{\begin{array}{c}{z}_{ijs_j}=<{\mu}_{z_{ijs_j}},{\eta}_{z_{ijs_j}},{\upsilon}_{z_{ijs_j}}>\mathrm{if}\kern0.5em {C}_{s_j}\ \mathrm{is}\ \mathrm{a}\ \mathrm{benefit}\ \mathrm{sub}\hbox{-} \mathrm{criterion}\\ {}{\left({z}_{ijs_j}\right)}^c=<{\upsilon}_{z_{ijs_j}},{\eta}_{z_{ijs_j}},{\mu}_{z_{ijs_j}}>\kern1em \mathrm{if}\kern0.5em {C}_{s_j}\ \mathrm{is}\ \mathrm{a}\ \mathrm{cost}\ \mathrm{sub}\hbox{-} \mathrm{criterion}\end{array}\right.,\\ {}i=1,\dots, m;j=1,\dots, n;{s}_j=1,\dots, {n}_j,\end{array}} $$

where ${r}_{ijs_j}$ denotes the normalized evaluation of the alternative *A*_*i*_ with respect to the sub-criterion ${C}_{s_j}$ of the criterion *C*_*j*_ given by the experts. It is necessary to invert the scale of the cost sub-criteria since a “no” (i.e., vote against) with regards to cost is actually positive. Thus, only experts’ evaluations with respect to cost sub-criteria are transformed by utilizing the complement operation.

**Step 3.2** Determine the picture fuzzy decision matrix *Q* = [*q*_*ij*_]_*m* × *n*_:

23 $$ {\displaystyle \begin{array}{l}\kern3.75em {C}_1\kern1.00em \cdots \kern0.5em \;{C}_n\\ {}Q=\begin{array}{c}{A}_1\\ {}\vdots \\ {}{A}_m\end{array}\left[\begin{array}{ccc}{q}_{11}& \cdots & {q}_{1n}\\ {}\vdots & \ddots & \vdots \\ {}{q}_{m1}& \cdots & {q}_{mn}\end{array}\right],\end{array}} $$

where ${q}_{ij}=<{\mu}_{q_{ij}},{\eta}_{q_{ij}},{\upsilon}_{q_{ij}}>$ is a PFN which represents aggregated normalized evaluation of the alternative *A*_*i*_ with respect to the criterion *C*_*j*_. It is calculated as follows:

24 $$ {\displaystyle \begin{array}{l}{q}_{ij}=<{\mu}_{q_{ij}},{\eta}_{q_{ij}},{\upsilon}_{q_{ij}}>={PFWGA}_{\delta}\left({r}_{ij1},\dots, {r}_{{ij n}_j}\right)=\underset{s_j=1}{\overset{n_j}{\otimes }}{\left({r}_{{ij s}_j}\right)}^{\delta_{s_j}}\\ {}=<\prod \limits_{s_j=1}^{n_j}{\left({\mu}_{r_{{ij s}_j}}+{\eta}_{r_{{ij s}_j}}\right)}^{w_j}-\prod \limits_{s_j=1}^{n_j}{\left({\eta}_{r_{{ij s}_j}}\right)}^{w_j},\prod \limits_{s_j=1}^{n_j}{\left({\eta}_{r_{{ij s}_j}}\right)}^{w_j},1-\prod \limits_{s_j=1}^{n_j}{\left(1-{\upsilon}_{r_{{ij s}_j}}\right)}^{w_j}>,\\ {}i=1,\dots, m;j=1,\dots, n,\end{array}} $$

where ${\delta}_j={\left({\delta}_1,\dots, {\delta}_{n_j}\right)}^T$ (*j* = 1, ..., *n*) is the weight vector of the sub-criteria of the criterion *C*_*j*_, with ${\delta}_{s_j}\in \left[0,1\right]$ and $\sum \limits_{s_j=1}^{n_j}{\delta}_{s_j}=1.$

**Step 3.3** Determine the picture fuzzy additive relative importance of each alternative as follows:

25 $$ {\displaystyle \begin{array}{l}{G}_i^{(1)}=<{\mu}_{G_i^{(1)}},{\eta}_{G_i^{(1)}},{\upsilon}_{G_i^{(1)}}>={PFWAA}_w\left({q}_{i1},\dots, {q}_{in}\right)=\underset{j=1}{\overset{n}{\oplus }}\left({w}_j\cdot {q}_{ij}\right)\\ {}=<1-\prod \limits_{j=1}^n{\left(1-{\mu}_{q_{ij}}\right)}^{w_j},\prod \limits_{j=1}^n{\left({\eta}_{q_{ij}}\right)}^{w_j},\prod \limits_{j=1}^n{\left({\eta}_{q_{ij}}+{\upsilon}_{q_{ij}}\right)}^{w_j}-\prod \limits_{j=1}^n{\left({\eta}_{q_{ij}}\right)}^{w_j}>,\kern0.75em i=1,\dots, m,\end{array}} $$

where *w* = (*w*_1_, …, *w*_*n*_)^*T*^ is the weight vector of the criteria, with *w*_*j*_ ∈ [0, 1] and $\sum \limits_{j=1}^n{w}_j=1.$.

**Step 3.4** Determine the picture fuzzy multiplicative relative importance of each alternative as follows:

26 $$ {\displaystyle \begin{array}{l}{G}_i^{(2)}=<{\mu}_{G_i^{(2)}},{\eta}_{G_i^{(2)}},{\upsilon}_{G_i^{(2)}}>={PFWGA}_w\left({q}_{i1},\dots, {q}_{in}\right)=\underset{j=1}{\overset{n}{\otimes }}{\left({q}_{ij}\right)}^{w_j}\\ {}=<\prod \limits_{j=1}^n{\left({\mu}_{q_{ij}}+{\eta}_{q_{ij}}\right)}^{w_j}-\prod \limits_{j=1}^n{\left({\eta}_{q_{ij}}\right)}^{w_j},\prod \limits_{j=1}^n{\left({\eta}_{q_{ij}}\right)}^{w_j},1-\prod \limits_{j=1}^n{\left(1-{\upsilon}_{q_{ij}}\right)}^{w_j}>,\kern0.75em i=1,\dots, m.\end{array}} $$

**Step 3.5** Determine the picture fuzzy joint generalized criterion of each alternative as follows:

27 $$ {G}_i=<{\mu}_{G_i},{\eta}_{G_i},{\upsilon}_{G_i}>={b}_1\cdot {G}_i^{(1)}\oplus {b}_2\cdot {G}_i^{(2)},\kern0.75em i=1,\dots, m, $$

where *b*_1_ and *b*_2_ denote the trade-off parameters of the WSM and WPM models, with *b*_1_, *b*_2_ ∈ [0, 1] and *b*_1_ + *b*_2_ = 1. If *b*_1_ = *b*_2_ = 0.5, then the WPS and WPM are equally appraised in the picture fuzzy WASPAS method. If the trade-off parameter *b*_1_ is 1 (i.e., *b*_2_ = 0), then the alternatives are ranked according to the WSM model. If the *b*_2_ value is 1, then the ranking follows the WPM model. According to the operational rules for the PFNs given in Definition 3 we have:

28 $$ {\displaystyle \begin{array}{l}{G}_i=<{\mu}_{G_i},{\eta}_{G_i},{\upsilon}_{G_i}>=<1-\left(1-{\mu}_{\Gamma_i^{(1)}}\right)\left(1-{\mu}_{\Gamma_i^{(2)}}\right),{\eta}_{\Gamma_i^{(1)}}{\eta}_{\Gamma_i^{(2)}},\\ {}\left({\eta}_{\Gamma_i^{(1)}}+{\upsilon}_{\Gamma_i^{(1)}}\right)\left({\eta}_{\Gamma_i^{(2)}}+{\upsilon}_{\Gamma_i^{(2)}}\right)-{\eta}_{\Gamma_i^{(1)}}{\eta}_{\Gamma_i^{(2)}}>,\kern0.75em i=1,\dots, m,\end{array}} $$

where:

29 $$ {\displaystyle \begin{array}{l}{\Gamma}_i^{(k)}=<{\mu}_{\Gamma_i^{(k)}},{\eta}_{\Gamma_i^{(k)}},{\upsilon}_{\Gamma_i^{(k)}}>={b}_k\cdot {G}_i^{(k)}\\ {}=<1-{\left(1-{\mu}_{G_i^{(k)}}\right)}^{b_k},{\left({\eta}_{G_i^{(k)}}\right)}^{b_k},{\left({\eta}_{G_i^{(k)}}+{\upsilon}_{G_i^{(k)}}\right)}^{b_k}-{\left({\eta}_{G_i^{(k)}}\right)}^{b_k}>,\kern0.75em k=1,2;i=1,\dots, m.\end{array}} $$

**Step 3.6** Calculate the defuzzified crisp (i.e., deterministic) value of the joint generalized criterion as follows:

30 $$ {S}_i={\mu}_{G_i}+\frac{\eta_{G_i}}{2}+\frac{\xi_{G_i}}{2}\left(1+{\mu}_{G_i}-{\upsilon}_{G_i}\right),\kern0.75em i=1,\dots, m. $$

where neutral degrees are distributed equally to positive and negative degrees. It is recommended to transform the picture fuzzy joint generalized criterion values into crisp values to simplify the final ranking step.

**Step 3.7** Rank the alternatives according to the decreasing crisp values of their joint generalized criterion. The highest value is the most desirable alternative.

## Case study

**Fig. 2:**
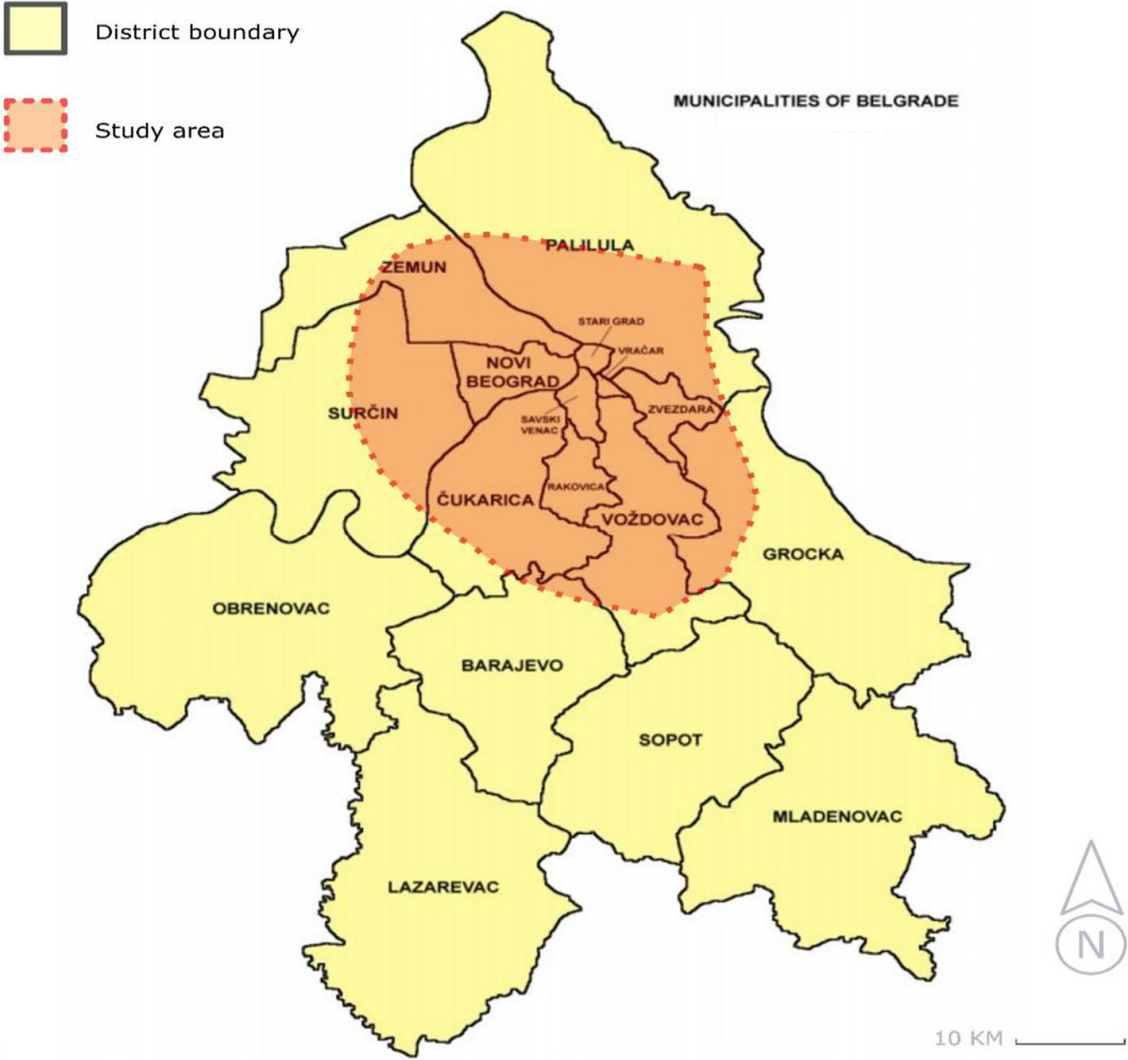
Study area

The capital of Serbia, Belgrade, is an industrial, economic, and traffic hub with a population of 1.69 million people [54]. The case study encompassed the inner-city area (Fig. 2). It incorporates the heavily urbanized central areas of Belgrade. The inner-city area covers an area of approximately 36,000 ha.

**Fig. 3:**
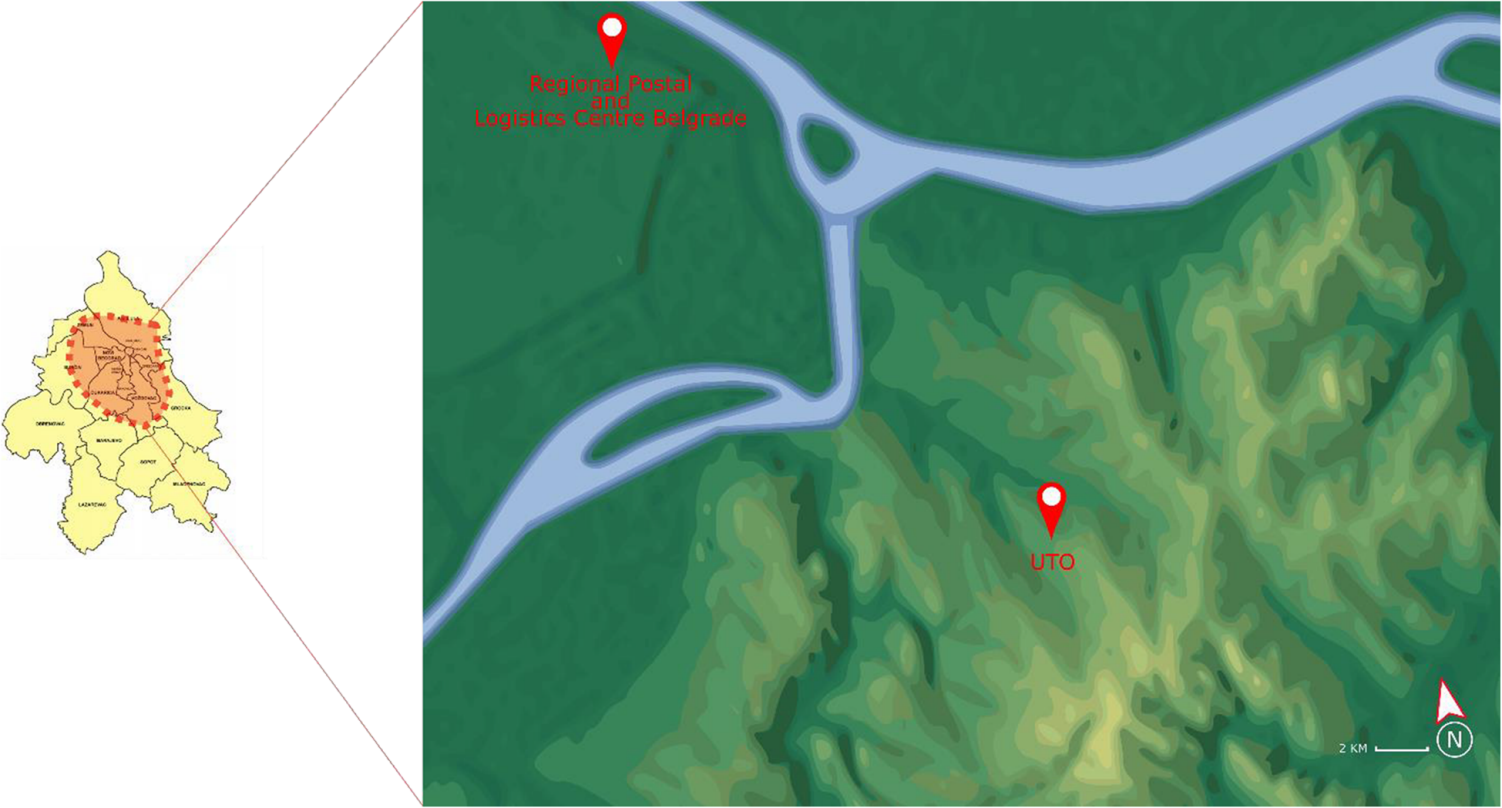
Terrain configuration in Belgrade

At present, in the inner-city area, the consignment delivery is conducted through the traditional LMD mode, while the delivery of other types of goods (primarily food) witnesses a new bicycle-based approach. The terrain configuration of the inner-city area in Belgrade is depicted in Fig. 3. It has a major role in defining alternatives and chances of implementing other LMD modes. Namely, the so-called “old town” characterizes a hilly terrain, where the streets are mainly downhill or uphill. Novi Beograd is separated from the old town by the Sava river and extremely flat.

**Fig. 4:**
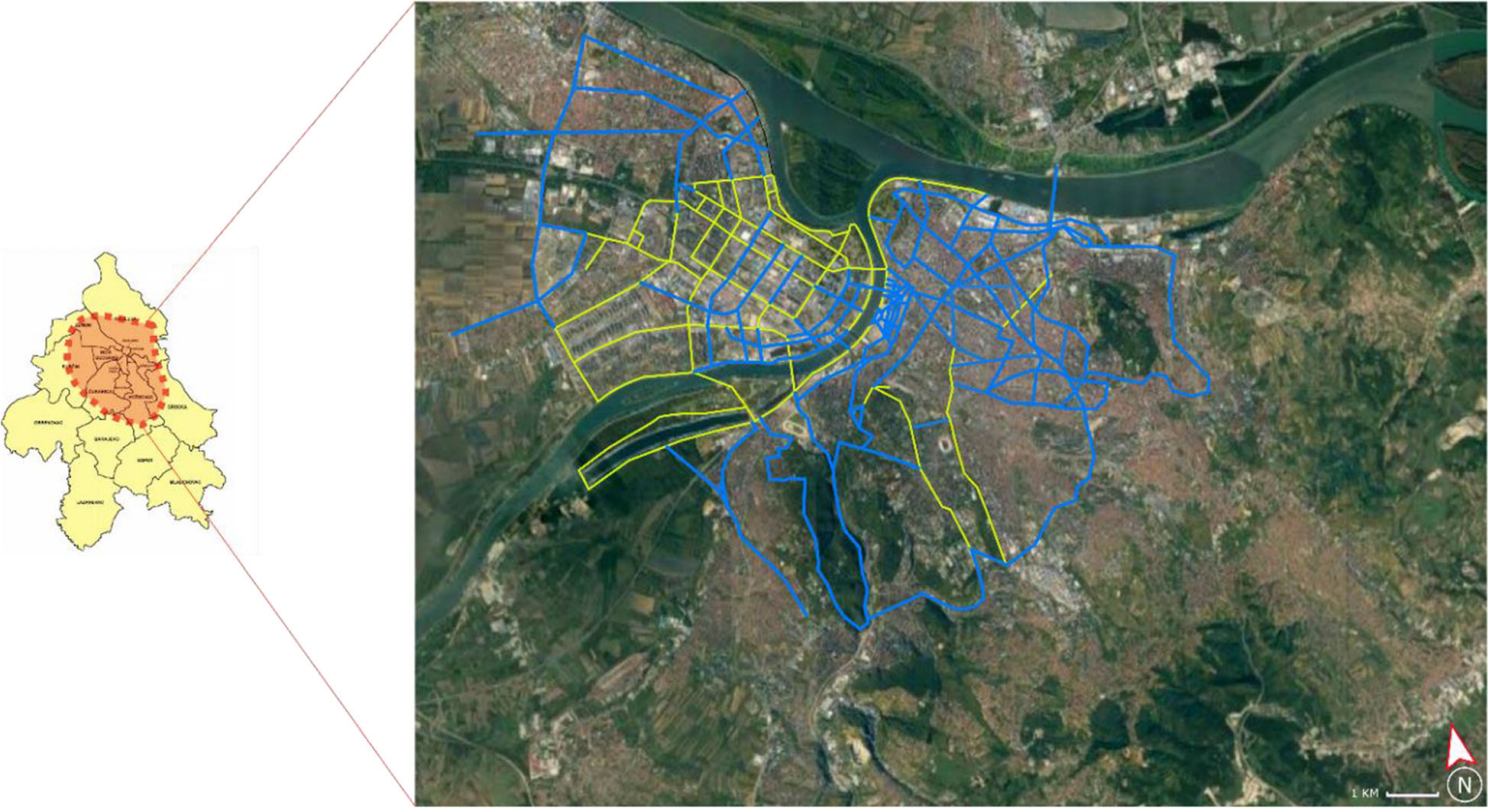
The network of bicycle lanes in Belgrade

When it comes to the infrastructure required for alternative LMD modes, there is a modest 65-km long network of bicycle lanes, which is spread in Novi Beograd (yellow-colored sections, Fig. 4). In the old town, bicycle lanes are mostly located within parks and recreational centers. The network is planned to be expanded by 300 km in the future (blue-colored sections, Fig. 4).

**Fig. 5:**
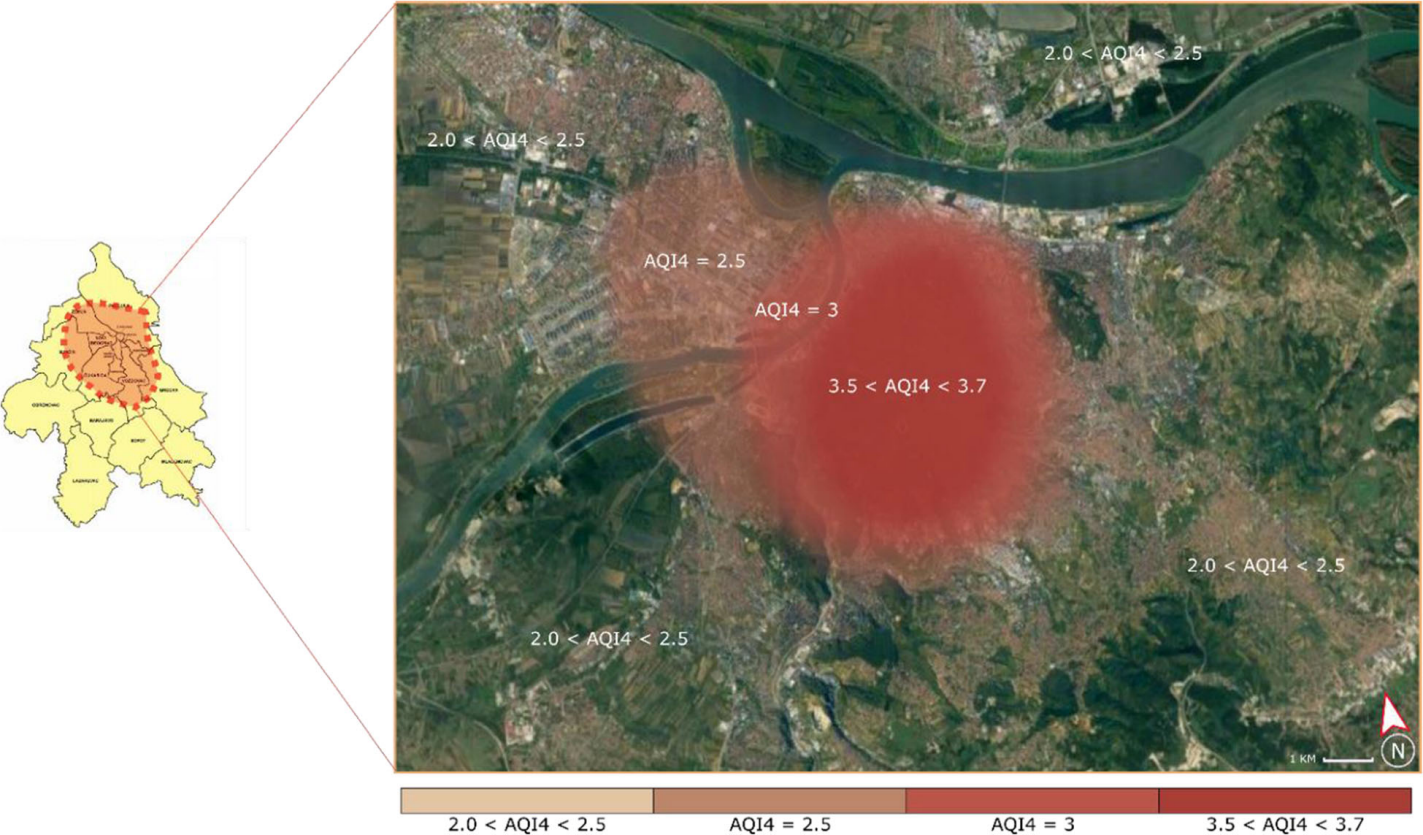
The air quality index in Belgrade – average values for the period from May 2019 to May 2020

Figure 5 shows the air quality index for sulfur dioxide, soot, nitrogen dioxide, and suspended particles (AQI4) in Belgrade. It is based on average values for the period from May 2019 to May 2020. From Fig. 5, it can be outlined that the air quality in all parts of Belgrade is very unhealthy (i.e., AQI4 > 2). Besides, on multiple occasions throughout 2019 and 2020, Belgrade was declared the city with the most polluted air in the world [23]. As a result, there is a strong motivation to introduce a more sustainable LMD mode since it could have an exceptionally positive impact on air quality in Belgrade. Besides, the paper aims to increase the general awareness of the impact of certain LMD modes on citizen lives and encourage managers to introduce environmentally friendly solutions. The concept of intermodality of different LMD modes is also possible.

The alternative modes specifically proposed for LMD in Belgrade are the following:

- (*A*_1_) *Traditional.* Postal companies mainly use the traditional LMD mode. In Belgrade, the couriers perform LMD most often by using a vehicle with a diesel engine (e.g., Peugeot Boxer and Partner). This delivery process involves several activities, such as loading shipments into a vehicle, driving to the LMD locations, and handing over a package.
- (*A*_2_) *Autonomous vehicle*. This is a very attractive LMD mode for delivery companies because it requires minor infrastructure adaptation and reduces courier engagement. The autonomous vehicle and drone LMD modes are similar. The main difference is the fact that autonomous vehicles move on the ground, especially on sidewalks or specially designed paths. The shipments are loaded in the storage space of an autonomous vehicle, and then it visits users’ locations.
- (*A*_3_) *Bicycle*. This LMD mode involves the use of cargo bicycles by a courier. It is particularly convenient from an environmental standpoint because it does not use any artificial energy source.
- (*A*_4_) *Drone*. The delivery process requires strong technological support. First, a drone with a shipment departs from a suitable station, which may be a fixed infrastructure object or a moving station within a suitable means of transport, located on the ground near a delivery location. Then, it is flying to the delivery location. Finally, the drone lands, recognizing the appropriate landing tag or by predefined coordinates, and leaves the shipment.
- (*A*_5_) *Postomate*. This LMD mode includes vending machines accessible to users 24/7, which are set up in typical locations such as gas stations, shopping malls, etc. They provide easy access authorization and fast picking up a shipment.
- (*A*_6_) *Tube transport*. This external transportation system for the delivery of shipments relies on an infrastructure made of pipes and specialized packaging for shipments. The shipments are transported through tubes to appropriate stations from which users take them.

It should be mentioned that each alternative could have certain limitations in exploitation, especially when shipments have inappropriate physical characteristics. For example, a bulky cargo (i.e., shipments of larger mass and volume) might be unsuitable for tube transport, drones, small autonomous vehicles, and bicycles. However, this is not an insurmountable obstacle to their widespread implementation in real-life delivery systems. Besides, the introduction of standardized shipments that meet the technical requirements and adequate price stimulations for users could present a possible solution strategy. On the other hand, a special portfolio of services with higher prices can be offered for bulky shipments since they have non-standard delivery requirements. Another limitation is related to the legal requirements imposed on postal operators. The most significant is the universal service obligation, which exists in almost all countries worldwide, and which implies, inter alia, the door-delivery to each user. For example, this can be seen as a limitation when introducing the means of out-of-home deliveries, such as postomates and tube transport. Accordingly, we introduce out-of-home delivery as possible alternatives in this paper; however, they should be considered as an additional choice for customers or added value service while a door delivery must stay as an option according to the legal requirements. Speaking about the carriers that are not universal service providers, the concept is similar; if they offer out-of-home delivery, it is just an additional possibility for the customers, not the exclusive solution. In this case, the reason is to fully satisfy all types of customers.

**Fig. 6:**
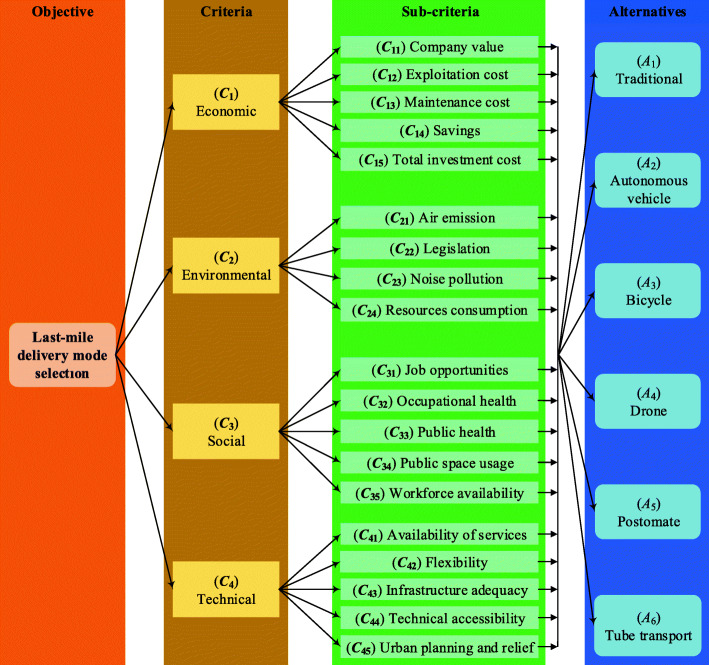
The hierarchical structure of the last-mile delivery mode selection problem

The study elucidated 19 sub-criteria for the selection of LMD mode in Belgrade. They were grouped into four criteria: economic, environmental, social, and technical. Figure 6 presents a hierarchical three-level structure of the highlighted problem.

The economic criterion (C_1_) has five sub-criteria:

- (*C*_11_) *Company value.* The impact on the value of a company through infrastructure and technical improvements, public acceptance, and brand strengthening.
- (*C*_12_) *Exploitation cost*. The cost incurred in exploiting the delivery concept; e.g., labor expenses, cost of fuel, and other consumables.
- (*C*_13_) *Maintenance cost*. The cost of maintaining an entire LMD mode so that all activities can be completed successfully.
- (*C*_14_) *Savings*. The savings that can be made by applying a particular LMD mode.
- (*C*_15_) *Total investment cost*. The necessary costs for implementing an LMD mode; e.g., construction of infrastructure, leasing space, procurement of technical equipment and software, etc.

The environmental criterion (C_2_) has four sub-criteria:

- (*C*_21_) *Air emission.* The environmental impact of an LMD mode through the level of air emissions exhausted during delivery.
- (*C*_22_) *Legislation.* The compliance of a delivery mode with available directives and environmental plans.
- (*C*_23_) *Noise pollution*. The impact on noise production is significant since delivery is realized in populated places in different parts of the day.
- (*C*_24_) *Resources consumption*. Raw materials and energy consumption of an LMD mode to provide services.

The social criterion (C_3_) has five sub-criteria:

- (*C*_31_) *Job opportunities*. The number, type (e.g., postal traffic, logistics, software engineering, etc.), and quality of jobs to implement an LMD mode.
- (*C*_32_) *Occupational health.* The impact of an LMD mode on workers’ health through direct contact.
- (*C*_33_) *Public health*. The expected and abrupt (e.g., explosions, fires, and other hazards) impacts on public health of an LMD mode.
- (*C*_34_) *Public space usage*. The occupation of public space by resources that are part of an LMD mode; e.g., usage of sidewalks and parking spaces.
- (*C*_35_) *Workforce availability*. A sufficient number of workers with proficiencies to implement and operate an LMD mode.

The technical criterion (C_4_) has five sub-criteria:

- (*C*_41_) *Availability of services*. The impact of an LMD mode on the spatial, temporal, and financial availability of services.
- (*C*_42_) *Flexibility*. The possibility of permanent adaptation to market changes.
- (*C*_43_) *Infrastructure adequacy*. The existence of adequate infrastructure (e.g., bicycle paths, drone stations, etc.) for an LMD mode.
- (*C*_44_) *Technical accessibility*. The existence of necessary technical equipment and means to implement an LMD mode.
- (*C*_45_) *Urban planning and relief*. Changes in urban planning and relief to implement and exploit an LMD mode.

Five relevant experts participated in this case study. These experts hold managerial positions in different postal companies and can influence the selection of LMD mode in Belgrade. Four experts are from the private sector and one expert comes from the public postal operator. Personal interviews were carried out with them to collect linguistic importance evaluations towards criteria, sub-criteria, and alternatives.

## Results and discussion

### Experimental results

***Phase 1:*** *information collection and representation*

**Step 1.1 **Linguistic evaluations given by five relevant experts towards six LMD modes in Belgrade are provided in Supplementary Table S1 in the Online Resource. The linguistic evaluation matrices are constructed with the help of Eq. (14).

**Step 1.2** The picture fuzzy evaluation matrices are given in Supplementary Table S2. They are determined based on the linguistic evaluation matrices (Supplementary Table S1) by applying Eq. (15). The picture fuzzy evaluations of six LMD modes are computed as the proportion of each item in the voting results.

**Step 1.3** Five relevant experts evaluate four criteria. Linguistic importance evaluations for the economic, environmental, social, and technical criteria are presented in Supplementary Table S3. Five linguistic criteria weight matrices are constructed with the help of Eq. (16).

**Step 1.4** Supplementary Table S4 presents the picture fuzzy criteria weight matrix. It is determined based on the linguistic criteria weight matrices (Supplementary Table S3) by applying Eq. (17). For instance, the picture fuzzy importance evaluation of the economic criterion (*C*_1_) given by five experts is ${v}_1=<{\mu}_{v_1},{\eta}_{v_1},{\upsilon}_{v_1}>=$ < 0.6, 0.4, 0 > (Supplementary Table S4). More detailed, from Supplementary Table S3 it can be seen that three experts have a positive attitude (i.e., vote “yes”) while two experts have a neutral attitude towards the criterion *C*_1_. As a result, the corresponding degree of positive membership is ${\mu}_{v_1}=\frac{3}{5}=0.6$ and the corresponding degree of neutral membership ${\eta}_{v_1}=\frac{2}{5}=0.4.$ The degrees of negative and refusal membership are 0 since there were no negative or invalid votes towards the economic criterion; i.e., ${\upsilon}_{v_1}={\xi}_{v_1}=0.$.

**Step 1.5** Linguistic importance evaluations towards the sub-criteria are presented in Supplementary Table S5. The corresponding matrices are constructed by using Eq. (18).

**Step 1.6** The picture fuzzy sub-criteria weight matrices are provided in Supplementary Table S6. They are determined based on the linguistic sub-criteria weight matrices (Supplementary Table S5) by applying Eq. (19).

***Phase 2:*** *determination of criteria and sub-criteria weights*

**Step 2.1 **The criteria weights are provided in Supplementary Table S4. They are calculated with the help of Eq. (20). The obtained weight vector of the criteria is *w* = (0.3774,  0.3302,  0.1038,  0.1887)^*T*^. Therefore, the criteria are ranked according to their importance as economic (*C*_1_), environmental (*C*_2_), technical (*C*_4_), and social (*C*_3_).

**Step 2.2 **By applying Eq. (21) the sub-criteria weights are calculated (Supplementary Table S6). According to this table, total investment cost (*C*_15_), air emission (*C*_21_), occupational health (*C*_32_), and infrastructure adequacy (*C*_43_), are the most important economic, environmental, social, and technical criteria, respectively.

***Phase 3:*** *decision-making*

**Step 3.1** The picture fuzzy normalized evaluation matrices for the LMD modes are presented in Supplementary Table S7. Only the experts’ evaluations with respect to cost sub-criteria are transformed by applying Eq. (22). Cost sub-criteria are exploitation cost (*C*_12_), maintenance cost (*C*_13_), total investment cost (*C*_15_), air emission (*C*_21_), noise pollution (*C*_23_), resources consumption (*C*_24_), occupational health (*C*_32_), public health (*C*_33_), public space usage (*C*_34_), and urban planning and relief (*C*_45_). The other nine sub-criteria are benefit type sub-criteria.

**Step 3.2** Supplementary Table S8 presents the picture fuzzy decision matrix of the considered problem. Aggregated normalized evaluations of six LMD modes with respect to each criterion are determined based on the sub-criteria weights (Supplementary Table S6) and the normalized evaluations (Supplementary Table S7) by applying Eq. (24). Supplementary Table S8 presents the picture fuzzy decision matrix of the considered problem. Aggregated normalized evaluations of six LMD modes with respect to each criterion are determined based on the sub-criteria weights (Supplementary Table S6) and the normalized evaluations (Supplementary Table S7) by applying Eq. (24).

**Steps 3.3˗3.4** The picture fuzzy additive relative importance and multiplicative relative importance of six LMD modes are computed by using the PFWAA operator (Eq. (25)) and PFWGA operator (Eq. (26)), respectively (Table 3).

**Table 3:** The additive and multiplicative relative importance, joint generalized criterion values, and ranks of the LMD modes

Alternative	Additive relative importance	Multiplicative relative importance	Joint generalized criterion		Rank
			Picture fuzzy	Crisp	
*A*_1_	< 0.303, 0.204, 0.392>	< 0.232, 0.204, 0.453>	< 0.268, 0.204, 0.422>	0.415	4
*A*_2_	< 0.183, 0.318, 0.374>	< 0.151, 0.318, 0.40>	< 0.167, 0.318, 0.387>	0.376	5
*A*_*3*_	< 0.439, 0.309, 0.159>	< 0.420, 0.309, 0.192>	< 0.430, 0.309, 0.175>	0.638	2
*A*_4_	< 0.304, 0.424, 0.185>	< 0.295, 0.424, 0.188>	< 0.30, 0.424, 0.187>	0.562	3
*A*_5_	< 0.506, 0.314, 0.125>	< 0.492, 0.314, 0.131>	< 0.499, 0.314, 0.128>	0.696	1
*A*_6_	< 0.171, 0.276, 0.459>	< 0.153, 0.276, 0.482>	< 0.162, 0.276, 0.470>	0.332	6

**Step 3.5 **The preferred value of the trade-off parameter *b*_1_ is 0.5 since it gives equal relative importance to additive and multiplicative relative importance of the alternatives. The picture fuzzy joint generalized criterion of each LMD mode (Table 3) is determined with the help of Eqs. (27)–(29).

**Step 3.6** The picture fuzzy joint generalized criteria of alternatives are defuzzified by applying Eq. (30). Table 3 provides the calculated crisp values.

**Step 3.7** Six alternative locations are ranked according to the decreasing crisp values of their joint generalized criterion (Table 3). The obtained ordering is *A*_5_ ≻ *A*_3_ ≻ *A*_4_ ≻ *A*_1_ ≻ *A*_2_ ≻ *A*_6_. The obtained results are reviewed by five invited experts. All experts confirmed the findings of this study. Also, they outlined that the proposed model gives a very meaningful ordering of the evaluated LMD modes.

### Ranking discussion

**Fig. 7:**
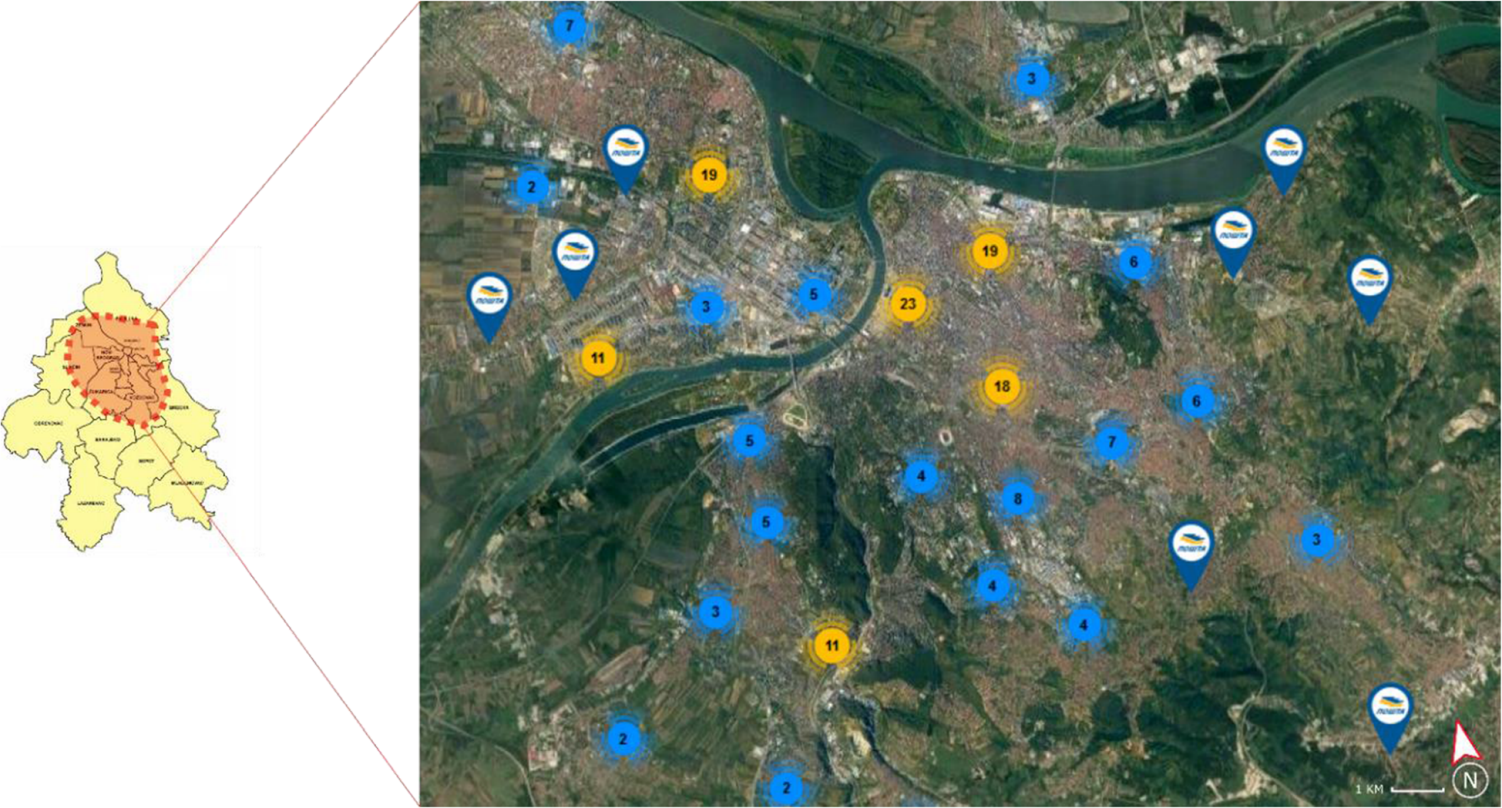
The network of locations belonging to the public postal operator in Belgrade

According to the presented picture fuzzy WASPAS method, “postomate” (*A*_5_) is the best LMD mode in the Belgrade scenario. One of the reasons that affected the positioning of *A*_5_ to the very top of the ladder is a well-developed network of postal operators and especially the public postal operator. Besides, the main drivers for the success of this LMD mode are less effort and lower cost to deliver parcels to postomates instead of users’ homes. Figure 7 shows the network of locations belonging to the network of the public postal operator in the inner-city area of Belgrade that is suitable for setting up postomates.

The bicycle LMD mode (*A*_3_) the second-best alternative. One of the main limitations of implementing this alternative in the Belgrade scenario is the terrain configuration (Fig. 3). Introducing an electric bicycle (e-bike) additionally contributes to how suitable this LMD mode is. Compared to a regular bicycle, the e-bike has more complex maintenance.

Drones and autonomous vehicles represent the future for LMD. At present, numerous limitations restrain their use. The technology that is necessary for their use is expensive and the legislation has not yet covered technological advancement in this area. Therefore, its commercial use in most countries would be problematic. Besides, there is no necessary infrastructure, which means that serious investments are needed in the very beginning.

The traditional LMD mode (*A*_1_) is still a fair solution, regardless of the negative environmental impact. The reason behind this is the mixed terrain configuration and years of harmonized functioning of this LMD in Belgrade. One of the ways to improve this mode is to replace existing vehicles with electric or hybrid ones.

Tube transport (*A*_6_) is the least preferred mode for LMD in Belgrade. It has not been proven suitable for the considered problem. Nevertheless, the analysis of this mode shows that it could be applied for the internal transport of shipments.

Upon initial review, the obtained results could be considered unexpected or even contradictory because the opinions of the experts from the industry do not correspond to the current situation in Belgrade, where mainly the traditional LMD mode is applied. The difference between the opinions of the experts and their actions in current practice might be explained by the following reasons:

i) The changing circumstances over time that lead to different decisions about LMD mode in different moments; i.e., the constant modifications in the technology price, infrastructure development, available funds for technology change, legislation, etc.
ii) An increasing number of users, the COVID-19 outbreak, severe traffic congestion problems, and the low air quality in Belgrade.
iii) The transformation between two LMD modes is mainly a long-lasting process.

Several examples are offered to illustrate the previously mentioned barriers/reasons why operators do not run their preferred delivery systems. First, the price of a certain new technology, such as drones and autonomous vehicles, is usually high at the beginning and it reduces over time, which very much influences the decision. Another example can be related to infrastructure development. Even some solutions can exist for years, the infrastructure needed for their employment is different in different moments. If we considered cargo bicycles, the cities should develop appropriate routes and this is usually a continuous process. In addition, the evolution of traditional cargo bicycles to e-bikes provides an opportunity to overcome unfavorable terrain. One more example can be related to the available funds of the operators provided for technology change. These funds change over time, accordingly affecting the decisions. Certain criteria that were neglected in the past might emerge and significantly affect the decision; e.g., the environmental impact. Taking into account its global significance, the rising environmental impact is expected, which will be reflected by favoring eco-friendly delivery methods. Finally, all carriers search for innovative LMD solutions; however, their implementation implies demanding planning and significant investments in the appropriate infrastructure and technology. Therefore, if the opinions of experts at the moment of the interview are different from the current practice, this can be considered reasonable and acceptable.

In our case study, four experts are from private postal companies characterized by a higher level of flexibility, where it is easier to decide on technology changes. Besides, one of these companies is currently in the process of implementing postomates in the city of Belgrade. On the other hand, one expert comes from the public postal operator, which is a state-owned company. As a rule, in such a system it is difficult to perform significant technology changes rapidly, primarily because decisions have to be verified by various bodies and managerial levels. Certainly, both private and public carriers tend to improve and innovate the infrastructure and process of shipment delivery.

New types of delivery bring an added value for customers by improving the quality of service. When it comes to the best-ranked alternative (i.e., postomates), the biggest contribution for the customers is the availability of service, namely they are 24/7 at their disposal. In this way, the customers can take over the shipment at any time during the day or night. In the literature, a confirmation about a huge users’ interest in 24/7 service availability can be found in Lazarević et al. [32]. Further, the customers see this aspect as one of the significant advantages of postomates [61]. Experiences are positive and users have confidence in their usage [33, 81]. The results of a pilot project concerning the application of postomates in Amsterdam indicate their higher cost-efficiency compared to the traditional delivery [62]. The positive aspect of the environmental sustainability of postomates has been confirmed through several papers [22, 44, 48]. Numerous studies that address location problems of postomates indicate the relevance and popularity of their application [12, 31, 71]. An additional enhancement in the postomates exploitation is the possibility of their outsourcing, providing also a possibility of sharing the same self-service machine by various companies dealing with delivery. Finally, a special convenience for the customers could be the possibility to choose the LMD mode according to their needs. This would imply a personalized approach for each customer that can be achieved by introducing an appropriate application for mobile phones.

### Sensitivity analyses

The sensitivity analyses to changes in the trade-off parameters and criteria weights are performed to check the robustness of the generated results.

The first sensitivity analysis explores how the trade-off parameter *b*_1_ of the presented picture fuzzy WASPAS method influences the ranking order of the LMD modes in the Belgrade scenario. The values of *b*_1_ are varied in the interval [0, 1] with an increment value of 0.1. In the base case, the value of the trade-off parameter *b*_1_ is 0.5. When the trade-off parameter *b*_1_ is 0, the LMD modes are ranked according to the WPM, and when *b*_1_ is 1, the MCDM problem is solved by the WSM. The same ranking results for the LMD mode selection problem are obtained in both extreme cases; i.e., the ordering is *A*_5_ ≻ *A*_3_ ≻ *A*_4_ ≻ *A*_1_ ≻ *A*_2_ ≻ *A*_6_ when evaluation of the alternatives is in accordance with the WPM and WSM. The ranks of all six alternatives are stable in the considered interval of *b*_1_ values. As a result, it is identified that the trade-off parameters do not affect the ranking of the alternative modes for LMD in Belgrade.

The second sensitivity analysis thoroughly investigates how the criteria weights influence the results. The scenarios are generated by changing the weight of the most important criterion while adjusting the weights of the other criteria as follows [59]:

31 $$ {w}_j^{\hbox{'}}=\left(1-{w}_t^{\hbox{'}}\right)\frac{w_j}{\left(1-{w}_t\right)},\kern1.00em j=1,\kern0.5em \dots, \kern0.5em \ n\kern0.24em \left|\;j\ne t\right., $$

where ${w}_j^{\hbox{'}}$ represents the adjusted weight of the criterion *C*_*j*_, ${w}_t^{\hbox{'}}$ is the adjusted weight of the most important criterion *C*_*t*_, *w*_*j*_ is the original weight of the criterion *C*_*j*_, and *w*_*t*_ is the original weight of the most important criterion *C*_*t*_.

**Fig. 8:**
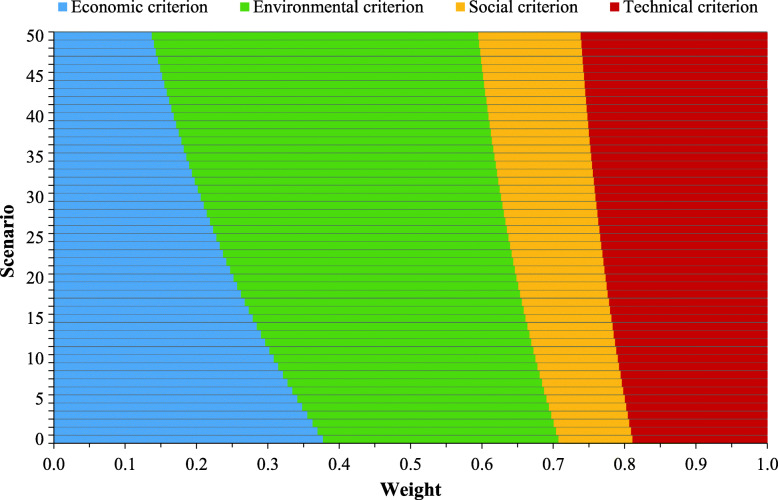
The simulated criteria weight scenarios

In the base scenario (scenario 0), the weight vector of the criteria is based on the criteria importance evaluations given by the invited experts; i.e., the weights of the economic, environmental, technical, and social criteria are *w*_1_ = 0.3774, *w*_2_ = 0.3302, *w*_3_ = 0.1038, and *w*_4_ = 0.1887, respectively (Fig. 8). Therefore, the most important is the economic criterion (*C*_1_). Fifty additional weight scenarios are simulated by reducing its weight with a rate of 2% while adjusting the weights of the other three criteria with the help of Eq. (31). The simulated weight scenarios are depicted in Fig. 8.

**Fig. 9:**
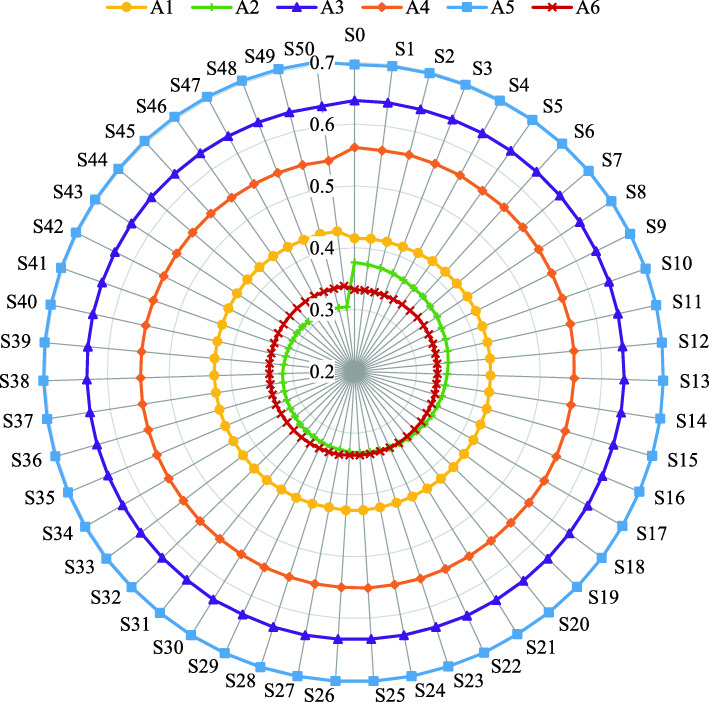
The sensitivity analysis to changes in the criteria weights

Figure 9 provides the joint generalized criterion values of the LMD modes in 50 simulated weight scenarios. Through all additional scenarios there is no change in the ranks of the first four alternatives; i.e., postomate (*A*_5_), bicycle (*A*_3_), drone (*A*_4_), and traditional LMD mode (*A*_1_). Minor changes in ranks occur with the remaining two alternatives (*A*_2_ and *A*_6_). More detailed, the initial ranks of the alternatives (i.e., *A*_5_ ≻ *A*_3_ ≻ *A*_4_ ≻ *A*_1_ ≻ *A*_2_ ≻ *A*_6_) are retained in scenarios 1–21, when the weight of the economic criterion is 0.25 < *w*_1_ ≤ 0.3774. For the weight of the most important criterion (*C*_1_) in the interval 0.1375 ≤ *w*_1_ ≤ 0.25, the fifth-ranked alternative *A*_2_ replaces its position with the alternative *A*_6_ and becomes the worst LMD mode.

According to the results of both sensitivity analyses, it can be outlined that the introduced picture fuzzy WASPAS method is highly robust. Besides, the LMD by postomates stands out as the best solution in the Belgrade scenario.

### Comparative analysis

Comprehensive comparative analysis with the available PFS-based MCDM methods can give a clear insight into how reliable are the obtained ranking results. For that reason, the investigated LMD mode selection problem in the Belgrade scenario is solved with the available PFS-based MCDM methods (Table 2). Table 4 shows a comparison of 11 PFS-based MCDM methods for solving the LMD mode selection problem. The developed method and the picture fuzzy MABAC, Cross-entropy, Projection, and PROMETHEE II methods have the perfect agreement between themselves since they generate the same ordering of the LMD mode alternatives in the Belgrade scenario.

**Table 4:** The comparative analysis of the picture fuzzy MCDM methods

Picture fuzzy MCDM method	Ordering
***WASPAS*** *(our study)*	A_5_ ≻ A_3_ ≻ A_4_ ≻ A_1_ ≻ A_2_ ≻ A_6_
**TOPSIS** [2, 60]	A_5_ ≻ A_3_ ≻ A_1_ ≻ A_4_ ≻ A_2_ ≻ A_6_
**EDAS** [35, 77]	A_5_ ≻ A_3_ ≻ A_4_ ≻ A_1_ ≻ A_6_ ≻ A_2_
**TODIM** [65, 69]	A_5_ ≻ A_3_ ≻ A_4_ ≻ A_2_ ≻ A_1_ ≻ A_6_
**VIKOR** [66]	A_5_ ≻ A_4_ ≻ A_3_ ≻ A_2_ ≻ A_6_ ≻ A_1_
**MABAC** [64]	A_5_ ≻ A_3_ ≻ A_4_ ≻ A_1_ ≻ A_2_ ≻ A_6_
**Cross-entropy** [68]	A_5_ ≻ A_3_ ≻ A_4_ ≻ A_1_ ≻ A_2_ ≻ A_6_
**Projection** [70]	A_5_ ≻ A_3_ ≻ A_4_ ≻ A_1_ ≻ A_2_ ≻ A_6_
**Grey relational projection** [27]	A_5_ ≻ A_3_ ≻ A_4_ ≻ A_2_ ≻ A_1_ ≻ A_6_
**Grey relational analysis** [36]	A_5_ ≻ A_3_ ≻ A_1_ ≻ A_4_ ≻ A_2_ ≻ A_6_
**PROMETHEE II** [58]	A_5_ ≻ A_3_ ≻ A_4_ ≻ A_1_ ≻ A_2_ ≻ A_6_

From Table 4 it can be seen that “postomate” (*A*_5_) represents the best-ranked alternative for the LMD in Belgrade through all picture fuzzy MCDM methods. The third alternative is ranked second in 10 out of 11 methods. Only the picture fuzzy VIKOR ranks “drone” (*A*_4_) as the second-best alternative. Eight methods put the LMD by drones in third place. The majority (six out of 11 methods) assess that the traditional LMD mode (*A*_1_) should take fourth place. The LMD by autonomous vehicles and tube transport are the two worst-ranked alternatives by the majority of the picture fuzzy MCDM methods. Tube transport (*A*_6_) is the worst alternative since nine out of 11 methods evaluate it as the least preferred mode for the LMD in Belgrade.

The preliminary analysis of the comparison results revealed high efficiency of the proposed picture fuzzy WASPAS method in evaluating LMD modes. However, quantitative metrics need to follow the comparative analysis to check the ranking similarity between different PFS-based MCDM methods. The similarity is investigated by using Spearman’s rank correlation coefficient (*rho*) and Kendall’s rank correlation coefficient (*tau-b*). Spearman’s rank correlation analysis can demonstrate the strength of the relationship of two PFS-based MCDM methods. Supplementary Table S9 provides the calculated *rho* values. According to this non-parametric test, the introduced PFS-based method has 95% of ranks matched. There are perfect relationships (*rho* = 1) between the picture fuzzy WASPAS method and the picture fuzzy MABAC, Cross-entropy, Projection, and PROMETHEE II methods. A very strong correlation (*rho* > 0.8) is another preferred outcome for the first consistency test. The developed PFS-based method has very strong relationships of the ranking results with the picture fuzzy TOPSIS, EDAS, TODIM, Grey relational projection, and Grey relational analysis methods. Besides, the first similarity test between the proposed method and the picture fuzzy VIKOR method can be seen as positive since a strong correlation (0.6 < *rho* ≤ 0.8) exists.

In the second consistency test, Kendall’s tau-b coefficient is applied to statistically analyze similarity in ranks obtained from different PFS-based MCDM methods. The computed *tau-b* values are given in Supplementary Table S9. According to Kendall’s rank correlation analysis, the picture fuzzy WASPAS method has 89% of ranks matched. The picture fuzzy WASPAS, MABAC, Cross-entropy, Projection, and PROMETHEE II methods generate the same ordering. The strengths of the relationships between the picture fuzzy WASPAS method and the fuzzy TOPSIS, EDAS, TODIM, VIKOR, Grey relational projection, and Grey relational analysis methods could be interpreted as strong since *tau-b* ≥ 0.5. Finally, according to two presented correlation analyses, it can be outlined that the presented picture fuzzy WASPAS method can generate highly consistent ranking results.

### Managerial implications

The picture fuzzy WASPAS method can be used to improve LMD in urban areas worldwide. The usage of this method can be beneficial to managers who are in charge of LMD. These managers, who deal with high levels of imprecise, vague, and uncertain information, can efficiently reveal the best LMD mode by applying the proposed method. In fact, in real-life decision-making, managers are usually divided into four groups of those who vote for, abstain, vote against, and refusal of voting. The voting mechanism is efficiently implemented in the presented decision-making framework. As a result, the picture fuzzy WASPAS method allows the managers to more naturally express their preferences over LMD modes by voting. Furthermore, the managers can also get additional insight into the stability of solutions by varying the trade-off parameters.

The presented methodological framework can directly assist managers to increase the availability of services and flexibility to market changes of LMD. Besides, the proposed PFS-based MCDM approach can reveal LMD modes whose implementation could endanger public and occupational health thus offering better medical crisis management; i.e., alternatives whose implementation supports the transition to a more sustainable society can be outlined by increasing the importance of the social criterion.

The study aids managers to differentiate relevant criteria of the LMD mode selection problem. It helps them in understanding the LMD mode assessment and selection process by elucidating four main criteria and 19 sub-criteria specifically related to the presented real-life case study. The presented PFS-based method could be applied to solve other emerging MCDM problems only by structuring them.

## Conclusions

An increasing number of users, medical crises (like the COVID-19 outbreak), traffic problems, and air pollution in urban areas worldwide have induced the need for companies in the postal and logistics industry to select a more sustainable LMD mode. The paper contributes practically by providing the computationally efficient method for solving the LMD mode selection problem since the implementation procedure is not complex as well as the picture fuzzy WASPAS method can be scaled to deal with any number of alternatives, evaluation criteria and sub-criteria, and experts with a small impact on the computing complexity. The presented methodological framework can directly assist managers to reveal LMD modes whose implementation could protect public and occupational health under medical crises thus providing the transition to a more sustainable society. Besides, it could aid managers to identify “greener” LMD modes thus supporting the transition to more sustainable cities. Furthermore, the constructed three-level hierarchical structure and elucidated evaluation criteria offer the practical framework for evaluating LMD modes in real-life applications.

The paper also contributes methodologically by extending the WASPAS method under the picture fuzzy environment. Its usage can be highly beneficial to managers who are in charge of LMD since it can take into account the neutral/refusal information and efficiently deal with high levels of imprecise, vague, and uncertain information. The sensitivity analyses to changes in the trade-off parameters and criteria weights confirmed the high robustness of the presented PFS-based MCDM method. The comparative analysis confirmed the high reliability of the developed picture fuzzy WASPAS method. The ranking similarity with the existing state-of-the-art picture fuzzy MCDM methods was analyzed by using Spearman’s and Kendall’s rank correlation coefficients. The rank correlation analyses confirmed the high consistency of the introduced picture fuzzy WASPAS method.

The provided real-life case study of Belgrade fully illustrated the potentials and applicability of the introduced PFS-based MCDM method. The LMD by postomates is the best alternative in the Belgrade scenario. This LMD mode can significantly increase the temporal and financial availability of services, offer better medical crisis management, and decrease traffic problems. In addition, a well-developed network of postomates could contribute to mitigating air pollution in Belgrade.

The PFS-based Direct rating method was used to determine criteria and sub-criteria weights. In future studies, the proposed picture fuzzy WASPAS method could be coupled with some other subjective criteria weighting methods such as AHP, BWM, and ANP. Finally, the proposed picture fuzzy WASPAS method can be used to solve other emerging MCDM problems.

## Supplementary Information

**Additional file 1: Table S1.** Linguistic evaluations for the LMD modes. **Table S2.** The picture fuzzy evaluation matrices for the LMD modes. **Table S3.** Linguistic importance evaluations for the criteria of the LMD mode selection problem. **Table S4.** The picture fuzzy criteria weight matrix and defuzzified values. **Table S5.** Linguistic importance evaluations for the sub-criteria of the LMD mode selection problem. **Table S6.** The picture fuzzy sub-criteria weight matrices and defuzzified values. **Table S7.** The picture fuzzy normalized evaluation matrices for the LMD modes. **Table S8.** The picture fuzzy decision matrix of the LMD mode selection problem. **Table S9.** The ranking similarity of the picture fuzzy MCDM methods.

## Data Availability

The datasets supporting the conclusions of this article are included within the article as its additional file.
