# Supplementary material for: Picture fuzzy WASPAS method for selecting last-mile delivery mode: a case study of Belgrade
Source: Eur Transp Res Rev. 2021 Jul 30;13(1):43. doi: 10.1186/s12544-021-00501-6 (PMC8323095; doi:10.1186/s12544-021-00501-6)
Supplement: Supplementary file 1 — Additional file 1: Table S1. Linguistic evaluations for the LMD modes. Table S2. The picture fuzzy evaluation matrices for the LMD modes. Table S3. Linguistic importance evaluations for the criteria of the LMD mode selection problem. Table S4. The picture fuzzy criteria weight matrix and defuzzified values. Table S5. Linguistic importance evaluations for the sub-criteria of the LMD mode selection problem. Table S6. The picture fuzzy sub-criteria weight matrices and defuzzified values. Table S7. The picture fuzzy normalized evaluation matrices for the LMD modes. Table S8. The picture fuzzy decision matrix of the LMD mode selection problem. Table S9. The ranking similarity of the picture fuzzy MCDM methods. [file 12544_2021_501_MOESM1_ESM.docx]

Supplementary material:

Picture fuzzy WASPAS method for selecting last-mile delivery mode:

A case study of Belgrade

**Table S1**

Linguistic evaluations for the LMD modes.

| **Alternative** | **DM** | **Sub-criterion** | | | | | | | | | | | | | | | | | | |
| --- | --- | --- | --- | --- | --- | --- | --- | --- | --- | --- | --- | --- | --- | --- | --- | --- | --- | --- | --- | --- |
|  |  | *C*_11_ | *C*_12_ | *C*_13_ | *C*_14_ | *C*_15_ | *C*_21_ | *C*_22_ | *C*_23_ | *C*_24_ | *C*_31_ | *C*_32_ | *C*_33_ | *C*_34_ | *C*_35_ | *C*_41_ | *C*_42_ | *C*_43_ | *C*_44_ | *C*_45_ |
| *A*_1_ | *D*_1_ | R | Y | N | R | R | Y | Y | Y | N | Y | Y | Y | Y | Y | Y | Y | Y | Y | Y |
|  | *D*_2_ | N | A | Y | N | A | Y | A | Y | Y | Y | A | R | Y | Y | Y | Y | A | Y | A |
|  | *D*_3_ | R | R | N | R | N | A | Y | Y | Y | Y | Y | Y | Y | Y | A | Y | Y | Y | Y |
|  | *D*_4_ | A | Y | A | N | R | Y | Y | Y | A | A | Y | A | A | Y | Y | A | Y | Y | Y |
|  | *D*_5_ | A | Y | Y | A | N | Y | Y | A | Y | Y | Y | Y | N | A | Y | Y | Y | A | Y |
| *A*_2_ | *D*_1_ | Y | N | A | Y | A | N | N | A | A | N | N | Y | R | R | A | N | N | R | N |
|  | *D*_2_ | Y | R | N | A | Y | A | A | R | Y | N | R | A | A | A | Y | N | A | N | A |
|  | *D*_3_ | A | A | Y | A | N | R | N | N | A | N | A | A | A | R | N | R | N | N | A |
|  | *D*_4_ | Y | A | A | Y | A | Y | N | Y | Y | A | N | R | N | A | A | A | N | A | A |
|  | *D*_5_ | Y | N | R | R | Y | Y | N | A | A | N | A | A | A | A | R | N | R | N | N |
| *A*_3_ | *D*_1_ | N | R | A | Y | N | R | Y | N | N | Y | Y | N | A | Y | A | Y | A | A | A |
|  | *D*_2_ | N | A | N | A | A | N | N | A | N | N | A | A | Y | Y | N | Y | N | Y | Y |
|  | *D*_3_ | N | N | R | Y | A | N | Y | A | R | A | Y | N | Y | Y | A | A | N | Y | Y |
|  | *D*_4_ | A | N | A | R | N | A | A | N | A | A | A | R | N | A | N | A | N | R | Y |
|  | *D*_5_ | N | A | A | Y | N | N | Y | N | N | Y | Y | N | Y | Y | A | N | A | Y | A |
| *A*_4_ | *D*_1_ | Y | N | A | A | N | N | N | A | N | N | N | Y | A | N | Y | A | A | N | N |
|  | *D*_2_ | Y | A | N | Y | A | A | R | R | N | A | A | A | R | A | Y | Y | N | N | Y |
|  | *D*_3_ | A | Y | A | Y | A | R | A | A | N | A | A | Y | N | N | N | A | Y | A | A |
|  | *D*_4_ | Y | N | Y | A | Y | A | A | Y | N | N | N | R | A | A | A | Y | A | R | Y |
|  | *D*_5_ | Y | A | A | Y | A | N | A | A | A | R | A | Y | N | N | Y | A | Y | A | Y |
| *A*_5_ | *D*_1_ | A | N | N | Y | N | N | Y | N | N | N | N | N | N | A | N | N | N | A | A |
|  | *D*_2_ | Y | A | A | Y | A | A | N | N | A | R | N | A | A | Y | A | N | Y | Y | N |
|  | *D*_3_ | N | N | R | N | N | N | A | N | R | N | A | N | R | A | Y | N | A | Y | A |
|  | *D*_4_ | N | A | A | A | A | N | Y | A | N | A | N | A | N | Y | R | A | A | Y | N |
|  | *D*_5_ | A | N | N | Y | Y | N | A | N | A | N | N | N | N | A | A | R | Y | Y | N |
| *A*_6_ | *D*_1_ | N | N | A | N | Y | N | N | R | N | A | N | N | N | A | A | N | N | A | N |
|  | *D*_2_ | A | A | R | N | A | A | A | N | A | R | N | A | N | N | N | N | N | A | A |
|  | *D*_3_ | Y | A | N | A | Y | A | N | N | N | N | A | A | N | N | N | N | N | N | N |
|  | *D*_4_ | R | R | A | R | Y | R | N | A | N | N | N | A | N | N | R | A | A | N | N |
|  | *D*_5_ | A | N | N | N | Y | N | N | N | R | A | A | R | A | R | N | N | N | N | R |

Decision-maker: DM; Yes: Y; Abstain: A; No: N; Refusal: R.

**Table S2**

The picture fuzzy evaluation matrices for the LMD modes.

| **Criterion** | **Sub-criterion** | **Alternative** | | | | | |
| --- | --- | --- | --- | --- | --- | --- | --- |
|  |  | *A*_1_ | *A*_2_ | *A*_3_ | *A*_4_ | *A*_5_ | *A*_6_ |
| *C*_1_ | *C*_11_ | <0, 0.4, 0.2> | <0.8, 0.2, 0> | <0, 0.2, 0.8> | <0.8, 0.2, 0> | <0.2, 0.4, 0.4> | <0.2, 0.4, 0.2> |
|  | *C*_12_ | <0.6, 0.2, 0> | <0, 0.4, 0.4> | <0, 0.4, 0.4> | <0.2, 0.4, 0.4> | <0, 0.4, 0.6> | <0, 0.4, 0.4> |
|  | *C*_13_ | <0.4, 0.2, 0.4> | <0.2, 0.4, 0.2> | <0, 0.6, 0.2> | <0.2, 0.6, 0.2> | <0, 0.4, 0.4> | <0, 0.4, 0.4> |
|  | *C*_14_ | <0, 0.2, 0.4> | <0.4, 0.4, 0> | <0.6, 0.2, 0> | <0.6, 0.4, 0> | <0.6, 0.2, 0.2> | <0, 0.2, 0.6> |
|  | *C*_15_ | <0, 0.2, 0.4> | <0.4, 0.4, 0.2> | <0, 0.4, 0.6> | <0.2, 0.6, 0.2> | <0.2, 0.4, 0.4> | <0.8, 0.2, 0> |
| *C*_2_ | *C*_21_ | <0.8, 0.2, 0> | <0.4, 0.2, 0.2> | <0, 0.2, 0.6> | <0, 0.4, 0.4> | <0, 0.2, 0.8> | <0, 0.4, 0.4> |
|  | *C*_22_ | <0.8, 0.2, 0> | <0, 0.2, 0.8> | <0.6, 0.2, 0.2> | <0, 0.6, 0.2> | <0.4, 0.4, 0.2> | <0, 0.2, 0.8> |
|  | *C*_23_ | <0.8, 0.2, 0> | <0.2, 0.4, 0.2> | <0, 0.4, 0.6> | <0.2, 0.6, 0> | <0, 0.2, 0.8> | <0, 0.2, 0.6> |
|  | *C*_24_ | <0.6, 0.2, 0.2> | <0.4, 0.6, 0> | <0, 0.2, 0.6> | <0, 0.2, 0.8> | <0, 0.4, 0.4> | <0, 0.2, 0.6> |
| *C*_3_ | *C*_31_ | <0.8, 0.2, 0> | <0, 0.2, 0.8> | <0.4, 0.4, 0.2> | <0, 0.4, 0.4> | <0, 0.2, 0.6> | <0, 0.4, 0.4> |
|  | *C*_32_ | <0.8, 0.2, 0> | <0, 0.4, 0.4> | <0.6, 0.4, 0> | <0, 0.6, 0.4> | <0, 0.2, 0.8> | <0, 0.4, 0.6> |
|  | *C*_33_ | <0.6, 0.2, 0> | <0.2, 0.6, 0> | <0, 0.2, 0.6> | <0.6, 0.2, 0> | <0, 0.4, 0.6> | <0, 0.6, 0.2> |
|  | *C*_34_ | <0.6, 0.2, 0.2> | <0, 0.6, 0.2> | <0.6, 0.2, 0.2> | <0, 0.4, 0.4> | <0, 0.2, 0.6> | <0, 0.2, 0.8> |
|  | *C*_35_ | <0.8, 0.2, 0> | <0, 0.6, 0> | <0.8, 0.2, 0> | <0, 0.4, 0.6> | <0.4, 0.6, 0> | <0, 0.2, 0.6> |
| *C*_4_ | *C*_41_ | <0.8, 0.2, 0> | <0.2, 0.4, 0.2> | <0, 0.6, 0.4> | <0.6, 0.2, 0.2> | <0.2, 0.4, 0.2> | <0, 0.2, 0.6> |
|  | *C*_42_ | <0.8, 0.2, 0> | <0, 0.2, 0.6> | <0.4, 0.4, 0.2> | <0.4, 0.6, 0> | <0, 0.2, 0.6> | <0, 0.2, 0.8> |
|  | *C*_43_ | <0.8, 0.2, 0> | <0, 0.2, 0.6> | <0, 0.4, 0.6> | <0.4, 0.4, 0.2> | <0.4, 0.4, 0.2> | <0, 0.2, 0.8> |
|  | *C*_44_ | <0.8, 0.2, 0> | <0, 0.2, 0.6> | <0.6, 0.2, 0> | <0, 0.4, 0.4> | <0.8, 0.2, 0> | <0, 0.4, 0.6> |
|  | *C*_45_ | <0.8, 0.2, 0> | <0, 0.6, 0.4> | <0.6, 0.4, 0> | <0.6, 0.2, 0.2> | <0, 0.4, 0.6> | <0, 0.2, 0.6> |

**Table S3**

Linguistic importance evaluations for the criteria of the LMD mode selection problem.

| **Criterion** | **Decision-maker** | | | | |
| --- | --- | --- | --- | --- | --- |
|  | *D*_1_ | *D*_2_ | *D*_3_ | *D*_4_ | *D*_5_ |
| *C*_1_ | Abstain | Yes | Yes | Yes | Abstain |
| *C*_2_ | Yes | Abstain | Abstain | Abstain | Yes |
| *C*_3_ | Refusal | No | No | Abstain | Refusal |
| *C*_4_ | Abstain | Abstain | Abstain | No | Abstain |

**Table S4**

The picture fuzzy criteria weight matrix and defuzzified values.

| **Criterion** | Degree of positive membership | Degree of neutral membership | Degree of negative membership | **Weight** |
| --- | --- | --- | --- | --- |
| *C*_1_ | 0.6 | 0.4 | 0 | 0.3774 |
| *C*_2_ | 0.4 | 0.6 | 0 | 0.3302 |
| *C*_3_ | 0 | 0.2 | 0.4 | 0.1038 |
| *C*_4_ | 0 | 0.8 | 0.2 | 0.1887 |

**Table S5**

Linguistic importance evaluations for the sub-criteria of the LMD mode selection problem.

| **Criterion** | **Sub-criterion** | **Decision-maker** | | | | |
| --- | --- | --- | --- | --- | --- | --- |
|  |  | *D*_1_ | *D*_2_ | *D*_3_ | *D*_4_ | *D*_5_ |
| *C*_1_ | *C*_11_ | Refusal | No | Refusal | Abstain | No |
|  | *C*_12_ | Yes | Abstain | Yes | Abstain | Yes |
|  | *C*_13_ | Yes | Abstain | No | Yes | Abstain |
|  | *C*_14_ | Abstain | No | Yes | Refusal | No |
|  | *C*_15_ | Yes | Yes | Yes | Abstain | Yes |
| *C*_2_ | *C*_21_ | Abstain | Yes | Yes | Yes | Abstain |
|  | *C*_22_ | Yes | Yes | Refusal | Refusal | Abstain |
|  | *C*_23_ | No | Abstain | Abstain | No | Yes |
|  | *C*_24_ | Yes | No | Abstain | No | No |
| *C*_3_ | *C*_31_ | Refusal | No | Refusal | Abstain | Refusal |
|  | *C*_32_ | Yes | Abstain | Abstain | Yes | Abstain |
|  | *C*_33_ | Abstain | Abstain | Yes | No | Yes |
|  | *C*_34_ | No | Yes | No | Abstain | No |
|  | *C*_35_ | Abstain | Refusal | No | Abstain | No |
| *C*_4_ | *C*_41_ | Yes | Yes | Abstain | Abstain | Abstain |
|  | *C*_42_ | Abstain | No | No | Yes | Abstain |
|  | *C*_43_ | Abstain | Yes | Abstain | Yes | Yes |
|  | *C*_44_ | Yes | Abstain | Abstain | Abstain | Abstain |
|  | *C*_45_ | No | Abstain | No | Refusal | Refusal |

**Table S6**

The picture fuzzy sub-criteria weight matrices and defuzzified values.

| **Criterion** | **Sub-criterion** | Degree of positive membership | Degree of neutral membership | Degree of negative membership | **Weight** |
| --- | --- | --- | --- | --- | --- |
| *C*_1_ | *C*_11_ | 0 | 0.2 | 0.4 | 0.0759 |
|  | *C*_12_ | 0.6 | 0.4 | 0 | 0.2759 |
|  | *C*_13_ | 0.4 | 0.4 | 0.2 | 0.2069 |
|  | *C*_14_ | 0.2 | 0.2 | 0.4 | 0.1310 |
|  | *C*_15_ | 0.8 | 0.2 | 0 | 0.3103 |
| *C*_2_ | *C*_21_ | 0.6 | 0.4 | 0 | 0.3509 |
|  | *C*_22_ | 0.4 | 0.2 | 0 | 0.3421 |
|  | *C*_23_ | 0.2 | 0.4 | 0.4 | 0.1754 |
|  | *C*_24_ | 0.2 | 0.2 | 0.6 | 0.1316 |
| *C*_3_ | *C*_31_ | 0 | 0.2 | 0.2 | 0.1545 |
|  | *C*_32_ | 0.4 | 0.6 | 0 | 0.3182 |
|  | *C*_33_ | 0.4 | 0.4 | 0.2 | 0.2727 |
|  | *C*_34_ | 0.2 | 0.2 | 0.6 | 0.1364 |
|  | *C*_35_ | 0 | 0.4 | 0.4 | 0.1182 |
| *C*_4_ | *C*_41_ | 0.4 | 0.6 | 0 | 0.2574 |
|  | *C*_42_ | 0.2 | 0.4 | 0.4 | 0.1471 |
|  | *C*_43_ | 0.6 | 0.4 | 0 | 0.2941 |
|  | *C*_44_ | 0.2 | 0.8 | 0 | 0.2206 |
|  | *C*_45_ | 0 | 0.2 | 0.4 | 0.0809 |

**Table S7**

The picture fuzzy normalized evaluation matrices for the LMD modes.

| **Criterion** | **Sub-criterion** | **Alternative** | | | | | |
| --- | --- | --- | --- | --- | --- | --- | --- |
|  |  | *A*_1_ | *A*_2_ | *A*_3_ | *A*_4_ | *A*_5_ | *A*_6_ |
| *C*_1_ | *C*_11_ | <0, 0.4, 0.2> | <0.8, 0.2, 0> | <0, 0.2, 0.8> | <0.8, 0.2, 0> | <0.2, 0.4, 0.4> | <0.2, 0.4, 0.2> |
|  | *C*_12_ | <0, 0.2, 0.6> | <0.4, 0.4, 0> | <0.4, 0.4, 0> | <0.4, 0.4, 0.2> | <0.6, 0.4, 0> | <0.4, 0.4, 0> |
|  | *C*_13_ | <0.4, 0.2, 0.4> | <0.2, 0.4, 0.2> | <0.2, 0.6, 0> | <0.2, 0.6, 0.2> | <0.4, 0.4, 0> | <0.4, 0.4, 0> |
|  | *C*_14_ | <0, 0.2, 0.4> | <0.4, 0.4, 0> | <0.6, 0.2, 0> | <0.6, 0.4, 0> | <0.6, 0.2, 0.2> | <0, 0.2, 0.6> |
|  | *C*_15_ | <0.4, 0.2, 0> | <0.2, 0.4, 0.4> | <0.6, 0.4, 0> | <0.2, 0.6, 0.2> | <0.4, 0.4, 0.2> | <0, 0.2, 0.8> |
| *C*_2_ | *C*_21_ | <0, 0.2, 0.8> | <0.2, 0.2, 0.4> | <0.6, 0.2, 0> | <0.4, 0.4, 0> | <0.8, 0.2, 0> | <0.4, 0.4, 0> |
|  | *C*_22_ | <0.8, 0.2, 0> | <0, 0.2, 0.8> | <0.6, 0.2, 0.2> | <0, 0.6, 0.2> | <0.4, 0.4, 0.2> | <0, 0.2, 0.8> |
|  | *C*_23_ | <0, 0.2, 0.8> | <0.2, 0.4, 0.2> | <0.6, 0.4, 0> | <0, 0.6, 0.2> | <0.8, 0.2, 0> | <0.6, 0.2, 0> |
|  | *C*_24_ | <0.2, 0.2, 0.6> | <0, 0.6, 0.4> | <0.6, 0.2, 0> | <0.8, 0.2, 0> | <0.4, 0.4, 0> | <0.6, 0.2, 0> |
| *C*_3_ | *C*_31_ | <0.8, 0.2, 0> | <0, 0.2, 0.8> | <0.4, 0.4, 0.2> | <0, 0.4, 0.4> | <0, 0.2, 0.6> | <0, 0.4, 0.4> |
|  | *C*_32_ | <0, 0.2, 0.8> | <0.4, 0.4, 0> | <0, 0.4, 0.6> | <0.4, 0.6, 0> | <0.8, 0.2, 0> | <0.6, 0.4, 0> |
|  | *C*_33_ | <0, 0.2, 0.6> | <0, 0.6, 0.2> | <0.6, 0.2, 0> | <0, 0.2, 0.6> | <0.6, 0.4, 0> | <0.2, 0.6, 0> |
|  | *C*_34_ | <0.2, 0.2, 0.6> | <0.2, 0.6, 0> | <0.2, 0.2, 0.6> | <0.4, 0.4, 0> | <0.6, 0.2, 0> | <0.8, 0.2, 0> |
|  | *C*_35_ | <0.8, 0.2, 0> | <0, 0.6, 0> | <0.8, 0.2, 0> | <0, 0.4, 0.6> | <0.4, 0.6, 0> | <0, 0.2, 0.6> |
| *C*_4_ | *C*_41_ | <0.8, 0.2, 0> | <0.2, 0.4, 0.2> | <0, 0.6, 0.4> | <0.6, 0.2, 0.2> | <0.2, 0.4, 0.2> | <0, 0.2, 0.6> |
|  | *C*_42_ | <0.8, 0.2, 0> | <0, 0.2, 0.6> | <0.4, 0.4, 0.2> | <0.4, 0.6, 0> | <0, 0.2, 0.6> | <0, 0.2, 0.8> |
|  | *C*_43_ | <0.8, 0.2, 0> | <0, 0.2, 0.6> | <0, 0.4, 0.6> | <0.4, 0.4, 0.2> | <0.4, 0.4, 0.2> | <0, 0.2, 0.8> |
|  | *C*_44_ | <0.8, 0.2, 0> | <0, 0.2, 0.6> | <0.6, 0.2, 0> | <0, 0.4, 0.4> | <0.8, 0.2, 0> | <0, 0.4, 0.6> |
|  | *C*_45_ | <0, 0.2, 0.8> | <0.4, 0.6, 0> | <0, 0.4, 0.6> | <0.2, 0.2, 0.6> | <0.6, 0.4, 0> | <0.6, 0.2, 0> |

**Table S8**

The picture fuzzy decision matrix of the LMD mode selection problem.

| **Alternative** | **Criterion** | | | |
| --- | --- | --- | --- | --- |
|  | *C*_1_ | *C*_2_ | *C*_3_ | *C*_4_ |
| *A*_1_ | <0.161, 0.211, 0.357> | <0.18, 0.20, 0.620> | <0.141, 0.20, 0.588> | <0.678, 0.20, 0.122> |
| *A*_2_ | <0.322, 0.380, 0.185> | <0.096, 0.261, 0.567> | <0.132, 0.445, 0.266> | <0.041, 0.261, 0.485> |
| *A_3_* | <0.395, 0.377, 0.115> | <0.606, 0.226, 0.073> | <0.322, 0.278, 0.363> | <0.192, 0.381, 0.398> |
| *A*_4_ | <0.370, 0.468, 0.162> | <0.260, 0.450, 0.109> | <0.110, 0.377, 0.354> | <0.335, 0.336, 0.266> |
| *A*_5_ | <0.467, 0.365, 0.128> | <0.622, 0.278, 0.073> | <0.481, 0.275, 0.132> | <0.338, 0.310, 0.227> |
| *A*_6_ | <0.130, 0.295, 0.471> | <0.243, 0.255, 0.423> | <0.301, 0.374, 0.171> | <0.028, 0.233, 0.683> |

**Table S9**

The ranking similarity of the picture fuzzy MCDM methods.

| **Picture fuzzy  MCDM method** | **Statistical**  **coefficient** | **Picture fuzzy MCDM method** | | | | | | | | | | | |
| --- | --- | --- | --- | --- | --- | --- | --- | --- | --- | --- | --- | --- | --- |
|  |  | **WASPAS** | **TOPSIS** | **EDAS** | **TODIM** | **VIKOR** | **MABAC** | **CE** | **Projection** | **GRP** | **GRA** | **PII** | **Overall** |
| ***WASPAS*** *(our study)* | *rho* | − | *0.943* | *0.943* | *0.943* | *0.771* | *1* | *1* | *1* | *0.943* | *0.943* | *1* | *0.95* |
|  | *tau-b* | − | *0.867* | *0.867* | *0.867* | *0.600* | *1* | *1* | *1* | *0.867* | *0.867* | *1* | *0.89* |
| **TOPSIS** | *rho* | 0.943 | − | 0.886 | 0.829 | 0.543 | 0.943 | 0.943 | 0.943 | 0.829 | 1 | 0.943 | 0.88 |
|  | *tau-b* | 0.867 | − | 0.733 | 0.733 | 0.467 | 0.867 | 0.867 | 0.867 | 0.733 | 1 | 0.867 | 0.80 |
| **EDAS** | *rho* | 0.943 | 0.886 | − | 0.829 | 0.714 | 0.943 | 0.943 | 0.943 | 0.829 | 0.886 | 0.943 | 0.89 |
|  | *tau-b* | 0.867 | 0.773 | − | 0.733 | 0.467 | 0.867 | 0.867 | 0.867 | 0.733 | 0.733 | 0.867 | 0.77 |
| **TODIM** | *rho* | 0.943 | 0.829 | 0.829 | − | 0.886 | 0.943 | 0.943 | 0.943 | 1 | 0.829 | 0.943 | 0.91 |
|  | *tau-b* | 0.867 | 0.773 | 0.733 | − | 0.733 | 0.867 | 0.867 | 0.867 | 1 | 0.733 | 0.867 | 0.83 |
| **VIKOR** | *rho* | 0.771 | 0.543 | 0.714 | 0.886 | − | 0.771 | 0.771 | 0.771 | 0.886 | 0.543 | 0.771 | 0.74 |
|  | *tau-b* | 0.600 | 0.467 | 0.467 | 0.733 | − | 0.600 | 0.600 | 0.600 | 0.733 | 0.467 | 0.600 | 0.59 |
| **MABAC** | *rho* | 1 | 0.943 | 0.943 | 0.943 | 0.771 | − | 1 | 1 | 0.943 | 0.943 | 1 | 0.95 |
|  | *tau-b* | 1 | 0.867 | 0.867 | 0.867 | 0.600 | − | 1 | 1 | 0.867 | 0.867 | 1 | 0.89 |
| **CE** | *rho* | 1 | 0.943 | 0.943 | 0.943 | 0.771 | 1 | − | 1 | 0.943 | 0.943 | 1 | 0.95 |
|  | *tau-b* | 1 | 0.867 | 0.867 | 0.867 | 0.600 | 1 | − | 1 | 0.867 | 0.867 | 1 | 0.89 |
| **Projection** | *rho* | 1 | 0.943 | 0.943 | 0.943 | 0.771 | 1 | 1 | − | 0.943 | 0.943 | 1 | 0.95 |
|  | *tau-b* | 1 | 0.867 | 0.867 | 0.867 | 0.600 | 1 | 1 | − | 0.867 | 0.867 | 1 | 0.89 |
| **GRP** | *rho* | 0.943 | 0.829 | 0.829 | 1 | 0.886 | 0.943 | 0.943 | 0.943 | − | 0.829 | 0.943 | 0.91 |
|  | *tau-b* | 0.867 | 0.733 | 0.733 | 1 | 0.733 | 0.867 | 0.867 | 0.867 | − | 0.733 | 0.867 | 0.83 |
| **GRA** | *rho* | 0.943 | 1 | 0.886 | 0.829 | 0.543 | 0.943 | 0.943 | 0.943 | 0.829 | − | 0.943 | 0.88 |
|  | *tau-b* | 0.867 | 1 | 0.733 | 0.733 | 0.467 | 0.867 | 0.867 | 0.867 | 0.733 | − | 0.867 | 0.80 |
| **PII** | *rho* | 1 | 0.943 | 0.943 | 0.943 | 0.771 | 1 | 1 | 1 | 0.943 | 0.943 | − | 0.95 |
|  | *tau-b* | 1 | 0.867 | 0.867 | 0.867 | 0.600 | 1 | 1 | 1 | 0.867 | 0.867 | − | 0.89 |

Cross-entropy: CE; Grey relational projection: GRP; Grey relational analysis: GRA; PROMETHEE II: PII.
